# Low-Salt Diet Reduces Anti-CTLA4 Mediated Systemic Immune-Related Adverse Events while Retaining Therapeutic Efficacy against Breast Cancer

**DOI:** 10.3390/biology11060810

**Published:** 2022-05-25

**Authors:** Durga Khandekar, Debolanle O. Dahunsi, Isaac V. Manzanera Esteve, Sonya Reid, Jeffrey C. Rathmell, Jens Titze, Venkataswarup Tiriveedhi

**Affiliations:** 1 Department of Biological Sciences, Tennessee State University, Nashville, TN 37209, USA; dkhandek@tnstate.edu; 2 Department Pathology, Microbiology and Immunology, Vanderbilt University Medical Center, Nashville, TN 37232, USA; debolanle.o.dahunsi@vumc.org (D.O.D.); jeff.rathmell@vumc.org (J.C.R.); 3 Department of Plastic Surgery, Vanderbilt University Medical Center, Nashville, TN 37232, USA; isaac.v.manzanera.esteve@vumc.org; 4 Division of Hematology and Oncology, Department of Medicine, Vanderbilt University Medical Center, Nashville, TN 37232, USA; sonya.reid@vumc.org; 5 Program in Cardiovascular and Metabolic Disorders, Duke-NUS Medical School, Singapore 169857, Singapore; jens.titze@duke-nus.edu.sg; 6 Division of Nephrology, School of Medicine, Duke University, Durham, NC 27710, USA; 7 Division of Pharmacology, Vanderbilt University, Nashville, TN 37240, USA

**Keywords:** breast cancer, immunotherapy, t-helper cells, cytokines, cancer biology

## Abstract

**Simple Summary:**

Immunotherapy has transformed breast cancer treatment. However, ICI-induced systemic inflammatory immune-related adverse events (irAE) remain a major clinical challenge. In our current communication, using breast tumor models, we demonstrated that a low salt diet could reduce irAE development following ICI therapy. Importantly, a low salt diet did not change the anti-tumor efficiency. Our current study provides a basis for future clinical trials to verify the role of a low salt diet in long-term immunotherapeutic efficiency in breast cancer patients.

**Abstract:**

Immune checkpoint inhibitor (ICI) therapy has revolutionized the breast cancer treatment landscape. However, ICI-induced systemic inflammatory immune-related adverse events (irAE) remain a major clinical challenge. Previous studies in our laboratory and others have demonstrated that a high-salt (HS) diet induces inflammatory activation of CD4+T cells leading to anti-tumor responses. In our current communication, we analyzed the impact of dietary salt modification on therapeutic and systemic outcomes in breast-tumor-bearing mice following anti-cytotoxic T-lymphocyte-associated protein 4 (CTLA4) monoclonal antibody (mAb) based ICI therapy. As HS diet and anti-CTLA4 mAb both exert pro-inflammatory activation of CD4+T cells, we hypothesized that a combination of these would lead to enhanced irAE response, while low-salt (LS) diet through blunting peripheral inflammatory action of CD4+T cells would reduce irAE response. We utilized an orthotopic murine breast tumor model by injecting Py230 murine breast cancer cells into syngeneic C57Bl/6 mice. In an LS diet cohort, anti-CTLA4 mAb treatment significantly reduced tumor progression (day 35, 339 ± 121 mm^3^), as compared to isotype mAb (639 ± 163 mm^3^, *p* < 0.05). In an HS diet cohort, treatment with anti-CTLA4 reduced the survival rate (day 80, 2/15) compared to respective normal/regular salt (NS) diet cohort (8/15, *p* < 0.05). Further, HS plus anti-CTLA4 mAb caused an increased expression of inflammatory cytokines (IFNγ and IL-1β) in lung infiltrating and peripheral circulating CD4+T cells. This inflammatory activation of CD4+T cells in the HS plus anti-CTLA4 cohort was associated with the upregulation of inflammasome complex activity. However, an LS diet did not induce any significant irAE response in breast-tumor-bearing mice upon treatment with anti-CTLA4 mAb, thus suggesting the role of high-salt diet in irAE response. Importantly, CD4-specific knock out of osmosensitive transcription factor NFAT5 using CD4cre/creNFAT5flox/flox transgenic mice caused a downregulation of high-salt-mediated inflammatory activation of CD4+T cells and irAE response. Taken together, our data suggest that LS diet inhibits the anti-CTLA4 mAb-induced irAE response while retaining its anti-tumor efficacy.

## 1. Introduction

Cancer immunotherapy utilizing immune-checkpoint inhibitors (ICI), such as, CTLA4 (cytotoxic T-lymphocyte-associated protein 4), PD1 (programmed cell death protein 1), and PDL1 (ligand for PD1), have revolutionized the field of cancer treatment [1]. Breast cancer is the second leading cause of cancer-related mortality in the US women [2]. Check-point inhibitors have been approved for high risk localized and metastatic triple negative breast cancers. In addition, ICI therapy is approved for patients with unresectable or metastatic, microsatellite instability-high (MSI-H), or mismatch repair deficient (dMMR) solid tumors that have progressed following prior treatment and those who have no satisfactory alternative treatment options [3,4,5]. Cancer remission following immunotherapy is achieved by the activation of the host immune responses. Various clinical studies have demonstrated that ICI therapy has obtained an overall response rate (ORR) of about 20–50% [6,7]. In fact, immunotherapy has modified the landscape of clinical practice in oncology in some intriguing and unimaginable ways. For example, tumor mutation burden (TMB) was historically associated with cancer resistance and chemo-failure. However, in the context of ICI efficiency, TMB induces a generation of multiple neoantigens, thus making TMB a favorable marker for immunotherapeutic success [8,9].

Since ICI’s mechanism of action relies on reversing the immune-brake phenomenon, which otherwise leads to inflammatory and autoimmune disorders, they often have off-target effects resulting in immune-related adverse events (irAE) causing the inflammation of diverse organs or tissues [10]. Life-threatening side-effects such as pneumonitis and myocarditis have been reported in up to 60% of cancer patients treated with ICI, requiring hospitalization and treatment with high doses of steroids and immunosuppressors [11,12]. Immune-related toxicities are most common in the first 3 months of treatment but sometimes can occur long after ICI has been discontinued. Interestingly, literature evidence has demonstrated a positive association between the development of irAE and anti-tumor responses to ICIs [13]. Both the anti-tumor immune response and irAE arise from enhanced host immune reaction, wherein activated pro-inflammatory CD4+T cells infiltrate both tumors and other healthy organs. Although irAE could indicate success of immunotherapy, the sheer fatal nature of this event makes it rather an unacceptable side-effect. Clinical methods to overcome irAE while retaining the anti-tumor efficacy of ICI are still unknown.

Chronic inflammation is a well-established hallmark of cancer playing a key role in different phases of cancer initiation and progression [14,15]. Inflammasome is a part of non-specific innate immune multimeric protein complex activated in response to pathogen-associated molecules and cellular stress [16]. To some extent, inflammasome activation is attributed in the pathogenesis of irAE [17]. Canonical inflammasome activation is associated with the formation of an oligomer proteolytic complex utilizing sensor proteins, including pyrin domain containing related protein family (NLRP3), leading to the upregulation of caspases 1 activity [18]. This proteolytic capability of caspase 1 causes the cleavage of inactive pro-interleukin (IL)-1β to active inflammatory IL-1β. In general, the activation of NLRP3-inflammasome causes a pro-inflammatory response arising from the release of inflammatory cytokines, popularly referred to as cytokine storm [19]. Various factors such as pathogen-associated oligonucleotide sequences, lipopolysaccharides (LPS), ion channel modulators, and metabolic and synthetic small molecules are known to activate inflammasome complex [20].

Several extrinsic and intrinsic factors have been implicated in the inflammatory activation of CD4+T cells. High-salt (HS) diet has been associated with the activation of CD4+T cells to pro-inflammatory Th1 and Th17 phenotype, along with the inhibition of anti-inflammatory Treg phenotype through the serum glucocorticoid kinase 1 (SGK1) pathway [21,22,23]. Indeed, HS diet has shown to reduce tumor progression in preclinical murine models through the activation of anti-tumor inflammatory immune responses to myeloid derived suppressor cells (MDSC) [24,25,26]. However, HS diet is also associated with the exacerbation of cardiovascular, autoimmune, and other chronic inflammatory diseases [27]. The impacts of an HS diet on the anti-cancer efficiency and off-target effects of ICI are not well understood [28,29]. The tonicity sensitive transcription factor, TonEBP, also called nuclear factor of activated T-cells 5 (NFAT5), is known to play a critical role in osmotic stress-mediated inflammatory responses in lymph nodes and spleen, leading to immune cell differentiation, activation and proliferation [30,31,32]. Previous studies in our laboratory have demonstrated that NFAT5 plays a critical role in the high-salt-mediated inflammatory activation of CD4+T cells [33]. Interesting, there are reports suggesting that osmotic stress could induce inflammasome activation [34,35]. However, in the context of tumor microenvironment, there is limited literature evidence on the role of HS diet on inflammasome activation. As both HS diet and ICI are known to induce the inflammatory activation of CD4+T cells, we hypothesized that the combination of these two would lead to an enhanced irAE response. Furthermore, we tested the possibility of low-salt (LS) diet in reducing the irAE response.

## 2. Materials and Methods

### 2.1. Murine Breast Tumor Model

Female C57Bl/6J mice were procured from Jackson Laboratories (Maine, ME, USA). Transgenic mice (CD4^cre/cre^NFAT5^flox/flox^ on C57Bl/6 background) with CD4-specific knock-out of NFAT5 were generated in Titze laboratory (referred to as CD4-NFAT5-KO) and kindly provided to us. Tail-snip genotyping was performed by PCR to test for the transgenic knock-out. All mice were housed in specific pathogen-free facilities at Vanderbilt University Medical Center in ventilated cages, with at most 5 animals per cage. The mice were provided ad libitum food and water until included in our experiments. All protocols followed the guidelines approved by the Vanderbilt Institutional Animal Studies Committee (Protocol#M1600078-00-AN2). For salt-modified diet experiments, mice were placed in three cohorts: (i) mice on a regular/ normal-salt diet, referred as the NS diet cohort (1% NaCl, cat#TD.90229, Envigo, Madison, WI, USA); (ii) mice on a high-salt diet, referred to as the HS diet cohort (4% NaCl, cat#TD.92034 with 1% NaCl supplemented water); and (iii) mice on a low-salt diet, referred as the LS diet cohort (0.1% NaCl, cat#TD.94268). For the HS and LS diet cohorts, the mice were put on a respective salt-adjusted diet two weeks prior to the injection of tumor cells, at which stage all mice in the three cohorts were pair-fed. The mice were injected with tumor cells at approximately 12 weeks of age. Py230 murine breast cancer cells (obtained from the American Type Culture Collection, Manassas, VA, USA) syngeneic for C57Bl/6 were cultured, trypsinized and washed with PBS (phosphate-buffered saline) before intra-mammary injection (0.75 × 10^5^ cells/mice) into the flank of the mice. The tumor volume was calculated using the formula V = (W^2^^ × L)/2 from caliper measurements, where V is tumor volume, W is tumor width, and L is tumor length. Appropriate mycoplasma free testing was performed on cell cultures to prevent contamination. The cells were cultured in RPMI1640 media in a 5% CO_2_ incubator along with media supplements such as fetal bovine serum, penicillin/streptomycin, fungizone, HEPES (4-(2-hydroxyethyl)-1-piperazineethanesulfonic acid) and L-glutamine, as recommended by the manufacturer and as previously described. For immunotherapy experiments, in vivo mAb anti-mouse IgG2b monoclonal antibodies (mAb) against CTLA-4 (CD152) (clone 9D9, Catalog#BE0164, BioXcell, Lebanon, NH, USA), along with the matched isotype control (clone MPC-11, Catalog#BE0086) were utilized. Mice were injected with 200 µg of mAb on days 7, 10 and 13. All chemicals were obtained either from Fisher Scientific (Waltham, MA, USA) or Sigma-Aldrich (St. Louis, MO, USA), unless mentioned otherwise. In accordance with Institutional Animal Care and Use Committee (IACUC)-approved protocol and guidelines from the Office of Laboratory Animal Welfare (OLAW), the following humane endpoint criteria requiring the sacrifice of the animals were adopted (would meet one of the following criteria of excessive pain, stress, injury or distress): (i) if the animal is impeded in its ability to ambulate; (ii) cannot obtain food or water; (iii) has 20% weight loss from starting body weight; (iv) tumor growth to above 1.5 cm in diameter; or (v) tumor ulceration.

### 2.2. CD4+T Cell Isolation

Single cell suspensions of tumor, lung, and peripheral blood mononuclear cells (PBMCs) were utilized for CD4+T cell isolation and purity analyzed by flow cytometry [33]. Single-cell suspensions were prepared from explanted murine breast tumors and lungs by digestion with a mixture of 0.1% collagenase type IV, 0.01% DNase I, and 2.5 U/mL hyaluronidase type V in HBSS (Hank’s balanced salt solution) for 2 h at room temperature. These cells were filtered and debris was removed through a 100 μm nylon mesh; then, they were washed and resuspended in HBSS for further studies. The tumor/lung infiltrating CD4+T cells were isolated from the breast tumors (or lungs, respectively) harvested from various experimental cohorts. Single-cell preparation was conducted using these harvested tissues. The CD4+T cells were isolated using immunomagnetic beads (MACS beads, Miltenyi Biotec, Sunnyvale, CA, USA) first by the depletion of CD8+T cells, and then with positive selection with CD4+T cells. The purified CD4+T cells were suspended in complete medium. For flow cytometry analysis, non-specific antibody binding was blocked by incubating cells with 12.5 μg/mL mouse IgG (12.5 μg/mL diluted in PBS on ice) for 30 min. The cells were then stained with FITC-labeled anti-mouse CD4-monoclonal antibodies (clone#GK1.5, Biolegend, San Diego, CA, USA). The rat IgG2b-FITC (clone#RTK4530, Biolegend, San Diego, CA, USA) was utilized as an isotype control. The CD4+T cell isolations with >95% purity from individual biological replicate were utilized further in our functional and molecular studies (Supplementary Figure S1). Data were acquired on BD LSR Fortessa flow cytometer (BD Bioscience, Franklin Lakes, NJ, USA) and analyzed using FlowJo V10 software (FlowJo LLC, Ashland, OR, USA).

For in vitro molecular and functional analysis, to determine the impact of hypertonic sodium chloride on naïve CD4+T cells, we utilized spleens harvested from wild type C57Bl/6. Briefly, spleen was harvested and minced to small 0.2 cm pieces. Single-cell splenocyte preparation was performed by enzymatic digestion with collagenase and DNase. The cell debris was removed with 100 μm nylon mesh followed by treatment with RBC lysis and cell wash (4 times) with HBSS buffer. The naïve CD4+T cells were isolated by magnetic bead isolation as mentioned above. The naïve CD4+T cells were treated for 3 days with Δ35 mM NaCl (or 35 mM sodium glutamate), anti-CD3 monoclonal antibody (mAb, 2 μg/mL) CD28 mAb (2 μg/mL) for 3 days, followed by the addition of IL-7 (10 ng/mL) and IL-2 (20 ng/mL) and incubation for an additional 4 days. For osmotic control, the cells were treated with 35 and 70 mM mannitol or urea used instead of Δ35 mM NaCl.

For NLRP3 knock down, two pooled NLRP3-specific siRNA were obtained from two independent vendors. In pooled-1 experiments, cells were transfected with either with mouse NLRP3 siRNA (Accel SMARTpool set of 4 siRNA, final concentration, 25 nM; cat. EQ-053455-00-0020, Dharmacon, Lafayette, CO, USA), or scrambled control siRNA (cat. D-001910-10-50, Dharmacon, Lafayette, CO, USA) were performed using Accell siRNA reagents provided by the manufacturer. In pooled-2 experiments, cells were transfected by either with mouse NLRP3 siRNA with a pool of 3 target-specific 19–25 nt siRNAs designed to knock down gene expression (Cat# sc-45470, final concentration, 25 nM, Santa Cruz Biotech, Dallas, TX, USA), or scrambled control siRNA (sc-37007) were performed using manufacturer-provided siRNA transfection reagent (sc-29528), transfection medium (sc-36868) and dilution buffer (sc-29527).

Similarly, for NFAT5 knock down, two pooled NFAT5-specific siRNA were obtained from two independent vendors. In pooled-1 experiments, cells were transfected with either mouse NFAT5 siRNA (Accel SMARTpool set of 4 siRNA, final concentration, 25 nM; cat. EQ-058868-00-0020, Dharmacon, Lafayette, CO, USA) or scrambled control siRNA (cat. D-001910-10, Dharmacon, Lafayette, CO, USA) using Accell siRNA reagents provided by the manufacturer. In pooled-2 experiments, cells were transfected either with mouse NFAT5 siRNA with a pool of 3 target-specific siRNAs for knock down (Cat# sc-38122, final concentration, 25 nM, Santa Cruz Biotech, Dallas, TX, USA), or scrambled control siRNA (sc-37007x) were performed by using manufacturer-provided reagents.

### 2.3. Real Time Quantitative Polymerase Chain Reaction (RT-qPCR)

The mRNA expression of genes in murine breast tumors and other tissues were analyzed using the TaqMan FAM-labeled RT-qPCR primers for murine NFAT5 (Mm00467257_m1), IFNγ (Mm00801778_m1), IL1β (Mm00434228_m1), NLRP3 (Mm00840904_m1), GADPH (Mm00467257_m1), and Actin (Mm02619580_g1); obtained from Applied Biosystems/Thermo Fisher Scientific (Grand Island, NY, USA) and utilized as per the manufacturer’s recommendation. Briefly, total RNA was extracted from 10^6^ cells using TRIzol reagent and analyzed as mentioned previously. RNA samples were quantified by absorbance at 260 nm. The RNA was reverse-transcribed, and RT-PCR (real time PCR) was performed in a final reaction volume of 20 μL using BioRad CFX96 (Hercules, CA, USA). Each sample was analyzed in four repeats and divide by a factor of four. Cycling conditions consisted of an initial denaturation of 95 °C for 15 min, followed by 40 cycles of 95 °C for 30 s, followed by 61 °C for 1 min. The final reaction volume of 50 µL was analyzed by 2^−ΔΔCq^ method [36].

### 2.4. Western Blot

Total proteins were extracted from single-cell suspension using lysis buffer as previously described [37]. Protein concentration was determined by Bradford assay (Bio-Rad, Philadelphia, PA, USA). Total proteins were electrophoretically separated on a 4–12% sodium dodecyl sulfate-polyacrylamide gradient gel (SDS-PAGE) and transferred onto a nitrocellulose membrane. The membranes were blocked overnight at 4 °C in phosphate-buffered saline (PBS) with 0.05% Tween 20 and probed by appropriate primary and HRP-labeled secondary antibodies. All primary and secondary Abs were obtained from Santa Cruz Biotech (Dallas, TX, USA) unless indicated otherwise. The following specific primary antibodies at 1:1000 dilution were utilized: NFAT-5 (sc-398171) and GADPH (sc-32233). The membrane was developed using the chemiluminescence kit (Millipore) and analyzed using AnalytikJena chemstudio instrument (Upland, CA, USA). Morphometric analysis was performed using the software provided by the company. Original full gels provided in supplementary data. 

### 2.5. Luminex

Initially, the protein concentration of the whole cell lysates was quantified by BCA Protein Assay Kit (Pierce Biotechnology/Thermo Fisher Scientific, Rockford, IL, USA), and 250 μg of protein was equalized in appropriate reagents and used per sample in the multiplex cytokine labeled magnetic beads (Bioplex Pro Mouse cytokine 23-plex, BioRad, Herculus, CA, USA) as per the manufacturer’s protocol [38]. The samples were analyzed on a BioPlex-200 instrument (Bio-Rad, Philadelphia, PA, USA) and data analyzed using BioPlex Manager^TM^ software. The median fluorescent intensity (MFI) was obtained with a detection target of 50 beads per region, low RP1 target for CAL2 calibration, and recommended doublet discriminator (DD) gates of 5000–25,000. The data were analyzed by comparing standard, control, and sample wells. A count of less than 37 beads was excluded from data analysis, as it is required us to reduce the potential outlier impact. Any points with a coefficient of variance (%CV) less than 25% and those with accuracy outside of 80–120% of the expected range were excluded from the standard curve. The analysis software was then used to fit a curve to this set of reliable standards using five-parameter logistic regression with default automated weighting fitted to at least 6 points. Cytokine concentrations were presented as mean ± SEM, (*) *p* < 0.05 (ANOVA, one-way with post hoc Bonferroni correction) compared with the NS cohort, (^$^) *p* < 0.05 (ANOVA, one-way with post hoc Bonferroni correction) compared with the HS cohort, and non-detectable indicates that the protein detection was below the minimum detection range for that particular cytokine.

### 2.6. Histochemical Staining

Lungs harvested at days 15 and 35 were fixed in 10% formaldehyde. Tissue sections were cut at 5 μm thickness, stained with H&E and analyzed under a Nikon ECLIPSE Ts2 microscope (Melville, NY) using NIS-Elements BR software (Melville, NY, USA) [39]. Morphometric calculations were performed using NIS-Elements BR software (Melville, NY, USA). The percentage of cellular infiltration was calculated at 5 different high power fields in H&E stained sections (40×), respectively. The data were represented as a mean ± SEM for *n* = 6, with each slide read at 5 different high power fields and divided by 5.

### 2.7. Sodium Magnetic Resonance Imaging

The live in vivo MRI in the breast-tumor-bearing murine models was performed at Vanderbilt University Institute of Imaging Sciences. The murine magnetic resonance images were acquired on a Bruker Biospec 15.2T with a horizontal bore and inner diameter (ID) = 110 mm and clear ID = 60 mm within the gradient coils. The RF coil of inner diameter (ID) and outer diameter (OD) at 36 and 59 mm, respectively, was a homemade dual-channel ^1^H/^23^Na volume birdcage. In vivo MRI acquisitions consisted of two different scans to generate proton and sodium images of the same regions of the mice. Proton MRI was acquired using a two-dimensional FLASH sequence with the following parameters: echo time/repetition time (TE/TR) = 2.37/100 ms; receiver bandwidth (BW) = 100 kHz; flip angle (FA) = 50°; field of view (FOV) = 40 × 40 mm^2^; number of acquisitions (NA) = 16; acquisition time (TA) = 3 min 24 sec; number of slices = 9; slice thickness = 1 mm; and matrix (MTX) = 128 × 128, which resulted in a real resolution of 0.3125 mm/pixel. Sodium images were obtained using a three-dimensional gradient echo (GE) sequence. The sequence parameters used in the sodium measurements were: TE/TR = 1.31/250 ms; BW = 15 kHz; FA = 90°; FOV = 40 × 40 × 16 mm^3^; NA = 38; TA = 40 min 32 sec; and MTX = 32 × 32 × 8 with zero filling to obtain a matrix of 64 × 64 × 8. The results were obtained as sodium images with a real resolution of 3.125 mm^3^/pixel and a nominal resolution of 0.78 mm^3^/pixel. The signal-to-noise ratios (SNR) for the in vivo mouse sodium images were calculated using the mean signal of a region of interest (ROI) divided by the standard deviation of the noise outside the mice. Cylindrical phantom tubes (two millimeter in diameter and six centimeters length) were positioned alongside each mouse. Each phantom was filled with water with different sodium concentrations (50, 60, 70, 80 and 90 mmol/L) providing a specific spectrum of intensities used to characterize sodium concentrations in the tumor.

### 2.8. Electrolyte and Tissue Osmolarity Measurements

Tissue osmolality was examined using a vapor-pressure osmometer (ELITechGroup, VAPRO Osmometer model 5600, Fisher Scientific, Hampton, NH, USA) as per the manufacturer’s recommendations. Briefly, a 10 µL serum sample was loaded to saturate SS-033 disc and tissue obtained from various mouse cohorts was placed in a 1.5 mL tube and snap-frozen by dry ice. The instrument was calibrated with the standard solutions provided by the manufacturer. For chemical analysis of tumor and serum, the concentrations of electrolytes sodium [Na^+^] and potassium [K^+^] were measured by flame atomic absorption spectrometry (Agilent 240FS AA, Santa Clara, CA, USA). The chloride (Cl^−^) content was measured by 0.1 N silver nitrate (AgNO_3_) titration (Titrando, MetrohmUSA, Fisher Scientific, Hampton, NH, USA). The tumor tissues were isolated and dissolved in 1% nitric acid (HNO_3_) for electrolyte analysis, as per the manufacturer’s recommendations.

### 2.9. Enzyme Linked Immunosorbent Assay (ELISA)

The circulating levels of c-reactive protein (CRP, kit#ab222511, Abcam, Waltham, MA, USA) and procalcitonin (kit#NBP2-81212, Novus Biological, Centennial, CO, USA) in the blood were quantitated by ELISA using commercial kits, as per the manufacturer’s protocol. The protein supernatant was diluted at 1:500 and quantified with a standard curve using the manufacturer-provided standards. Detection at 450 nm was performed using EMax Plus spectrophotometer, and data analysis was carried out using software provided by the manufacturer (Molecular Devices, Sunnyvale, CA, USA).

### 2.10. Caspase 1 Activity Assay

Caspase1 activity was assessed using the Caspase-Glo 1 Inflammasome Assay (catalog#G9953, Promega, Madison, WI, USA), performed as per the manufacturer’s protocol. Briefly, the cell lysate was treated with 5 × 10^4^ cells with caspase 1 specific substrate, Z-WEHD-aminoluciferin at a final concentration of 20 μM, along with the proteosomal inhibitor MG-132 (provided by manufacturer). The mixture was incubated at 37 °C followed by the addition of the luciferase substrate. The luminescence was read using FilterMax F3 multi-mode plate reader (Molecular Devices, Sunnyvale, CA, USA).

### 2.11. Statistical Analysis

Data were expressed as the mean ± SEM (standard error of mean). Significant differences between groups were assessed using Tukey HSD pair-wise comparisons for two groups and one-way ANOVA with post hoc Bonferroni correction or one-way ANOVA with Dunnett’s test for multiple comparisons. A *p*-value of <0.05 was considered significant. All data analysis was obtained using Origin 6 software (Origin Labs, Northampton, MA, USA) or SPSS software, version 21 (IBM corporation, Armonk, NY, USA).

## 3. Results

### 3.1. Tumor and Plasma Electrolyte Changes following Salt Modified Diet

**Figure 1:**
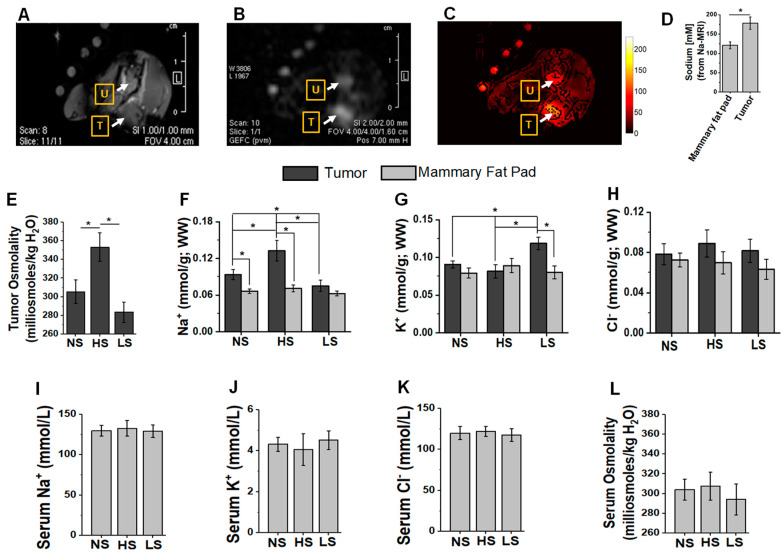
Sodium MRI in vivo live imaging of Py230-C57Bl/6 breast tumor. Py230 breast tumor cells were injected into right mammary fat pad in 8-week-old female C57Bl/6 mice. The imaging was performed on day 35 post-injection. (**A**) Proton-MRI; (**B**) Sodium(Na)-MRI; (**C**) heat map semi-quantitation of [Na^+^]. ‘T’ refers to orthotopic breast tumor; ‘U’ refers to urinary bladder (also serves as internal positive control as [Na^+^] is generally high in urine); (**D**) quantitative determination of [Na^+^] concentration from Na-MRI (*n* = 4). mean ± SEM, (*) *p*-value < 0.05, data analyzed by one-way ANOVA. Next, we determined tumor sodium accumulation following dietary salt (NaCl) modulation. (**E**) Tumor osmolality changes analyzed by vapor pressure osmometer following dietary salt modulation. (**F**–**H**) Tumor and contralateral mammary fat pad [Na^+^], [K^+^], and [Cl^−^] concentrations (mmol/g, WW; where WW refers to wet weight) following regular/normal-salt (NS, 1% NaCl), high-salt (HS, 4% NaCl) and low-salt (LS, 0.1% NaCl) diet; [Na^+^] and [K^+^] measured by flame atomic absorption, and [Cl^−^] analyzed by silver nitrate titration. (**I**–**L**) Plasma electrolyte and osmolality changes following dietary salt modification. (**E**–**L**) Data analyzed by Tukey HSD pair-wise comparison and one-way ANOVA for multiple comparisons; data presented as mean ± SEM, *n* = 8 per cohort, (*) *p*-value < 0.05.

To determine the tumor [Na^+^] concentration, we utilized live in vivo Na^23^-MRI in C57Bl/6 mice injected with Py230 breast cancer cells (on day 35 post-injection). As shown in Figure 1A–D, Na^23^-MRI studies suggested a 50–70% increase in ionic sodium [Na^+^] concentration compared to the surrounding soft tissue. It is of interest to note that these data are in agreement with literature evidence from human studies from other investigators, who have also shown a 30–70% increase in [Na^+^] concentration in human breast cancer patients [40,41,42]. To quantitatively determine the intra-tumoral electrolyte concentrations, sodium and potassium ions were analyzed by flame atomic absorption, chloride by silver nitrate titration, and tumor osmolality by vapor pressure osmometer. As shown in Figure 1E–H, the HS diet increased the breast tumor osmolality (353 ± 15 mOsm/kg H_2_O) as compared to the NS diet cohort (306 ± 13 mOsm/kg H_2_O, *p* < 0.05). There was slight decrease (statistically insignificant from NS diet) in tumor osmolality following LS diet treatment (283 ± 11 mOsm/kg H_2_O). The [Na^+^] concentration in breast tumors from mice fed on NS diet was significant higher compared to the contralateral mammary fat pad tissue in the same mice (NS tumor: 0.094 ± 0.008 mmol/g, WW; vs. fat pad: 0.066 ± 0.004 mmol/g, WW, *p* < 0.05). Further, [Na^+^] concentration in the HS diet cohort (0.133 ± 0.017 mmol/g, WW, *p* < 0.05) was significantly higher compared to the NS diet cohort, while the LS diet cohort (0.075 ± 0.009 mmol/g, WW) had significantly lower tumor [Na^+^] concentration compared to both NS diet (*p* < 0.05) and HS diet (*p* < 0.05) cohorts. Interestingly, tumor [K^+^] concentration was higher in the LS diet cohort compared to both NS diet (*p* < 0.05) and HS diet cohorts (*p* < 0.05). However, tumor [Cl^−^] concentration was similar between all three cohorts. Whereas dietary salt modification changed tumor sodium and osmolality concentrations, there was no statistically significant difference between serum osmolality and electrolyte concentrations among all three cohorts (Figure 1I–L). These data suggest that dietary salt modification plays a critical role in modulating the intrinsic ability of the tumor to accumulate sodium.

### 3.2. Anti-CTLA4 mAb Treatment in Low-Salt-Diet Cohort Enhanced the Survival

**Figure 2:**
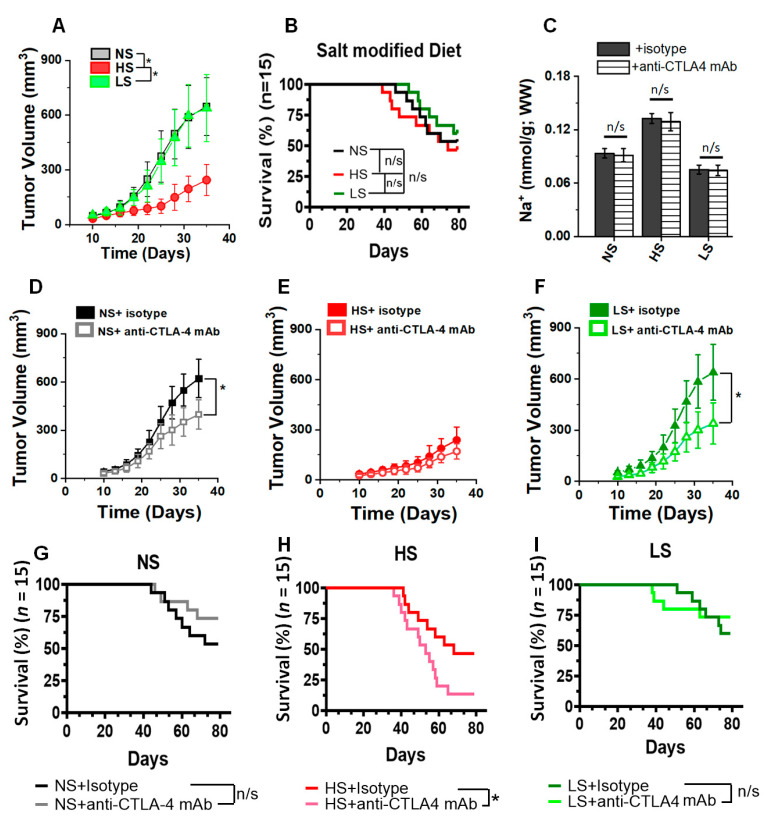
Tumor volume kinetics and survival analysis following salt-modified diet. (**A**) Tumor volume changes following dietary salt modification (NS, normal/regular salt; HS, high salt; LS, low salt); tumor volume kinetics followed for 5 weeks. Data presented as mean ± SEM, *n* = 8, statistical analysis was performed using two-way ANOVA multiple comparisons, Dunnet’s post-test (*) *p*-value < 0.05. (**B**) Survival rate in the three salt modified diet cohorts, tumor-bearing mice followed for 80 days. The *n* = 15 per cohort, statistical analysis performed by log-rank Mantel–Cox comparison. (**C**) [Na^+^] concentration in the murine breast tumors with isotype vs. anti-CTLA4 mAb treatment. Data analyzed by post hoc Bonferroni correction, n/s = not significant. (**D**–**F**) Tumor progression kinetics in NS (**D**), HS (**E**) and LS (**F**) cohorts following treatment with anti-CTLA4 monoclonal antibody (mAb) or isotype control with 200 µg injected intraperitoneally (i.p.) on days 7, 10 and 13, and followed for 5 weeks. Data presented as mean ± SEM, *n* = 8, statistical analysis was performed using multiple *t*-test, (*) *p*-value < 0.05. (**G**–**I**) Survival rate analysis in NS (**G**), HS (**H**) and LS (**I**) cohorts following treatment with anti-CTLA4 monoclonal antibody (mAb) or isotype control with 200 µg on days 7, 10 and 13, and followed for 80 days. The *n* = 15 per cohort, statistical analysis performed by log-rank Mantel–Cox comparison. Hazard ratio (HR) was performed by Mantel–Haenszel method.

To determine the impact of salt-modified diet on breast tumor progression, we utilized a Py230-C57Bl/6 murine breast cancer model. As shown in Figure 2A, the HS diet reduced tumor progression (day 35, HS: 244 ± 86 mL vs. NS: 647 ± 158 mL, *p* < 0.05), while the LS diet did not significantly change tumor progression (638 ± 184 mL, *p* = n/s). These data are in good agreement with previous evidence from other investigators showing that HS diet reduced tumor progression, which was ascribed to the HS-diet-mediated inflammatory activation of immune cells [24,25]. Survival analysis in tumor-bearing mice demonstrated that (Figure 2B) the low-salt-diet cohort had slightly higher survival (but statistically insignificant) compared with the high-salt-diet cohort (day 80, LS: 9/15, HS: 7/15, *p* = n/s; hazard ratio, HR: 2.13, 95% CI: 0.56–6.93); however, compared with the normal-salt diet, neither (high- nor low-salt) cohorts showed a statistically significant survival benefit. Next, we determined the impact of salt diet modification on the anti-tumor efficiency of anti-CTLA4 mAb based ICI therapy in our murine breast cancer model. Further, it is to be noted that treatment with anti-CTLA4 mAb did not change the tumor [Na^+^] concentration (Figure 2C) in the respective salt-matched cohorts. An analysis of tumor progression kinetics demonstrated that anti-CTLA4 mAb treatment reduced the tumor volume in NS (Figure 2D) and LS (Figure 2F) diet cohorts, as compared to respective isotype controls. As noted in Figure 2E, as HS diet by itself reduced the tumor growth, co-treatment with anti-CTLA4 mAb in this cohort did not show any significant decrease in tumor volume. Further, we did not notice any significant decrease in tumor progression in the anti-CTLA4 mAb-treated LS diet cohort (Figure 2F), as compared with the NS plus anti-CTLA4 mAb cohort (Figure 2D). Following anti-CTLA4 mAb treatment (Figure 2H), there was a marked decrease in survival in the high-salt-diet cohort (day 80, HS+isotype: 7/15, HS+anti-CTLA4 mAb: 2/15, *p* < 0.05; HR: 0.38, 95% CI: 0.16–0.93). There was no statistically significant survival advantage in NS (Figure 2G) and LS (Figure 2I) diet cohorts as compared to the respective with and without anti-CTLA4 therapy cohorts. Importantly, there was a significant survival advantage in the LS diet plus anti-CTLA4 mAb cohort (Figure 2I) when compared to the NS diet plus anti-CTLA4 mAb cohort (Figure 2G, *p* = n/s). In line with these survival data, the HS plus anti-CTLA4 showed up to a 17% weight loss (day 80) compared with the HS plus isotype control, suggesting that the decreased survival could be due to systemic inflammatory responses (Supplementary Figure S2). Taken together, these data demonstrated that tumor-bearing mice had a better survival when put on an LS diet and co-treated with immune checkpoint therapy.

### 3.3. Low-Salt Diet Reduced Anti-CTLA4-Mediated Inflammatory Lung Infiltration

**Figure 3:**
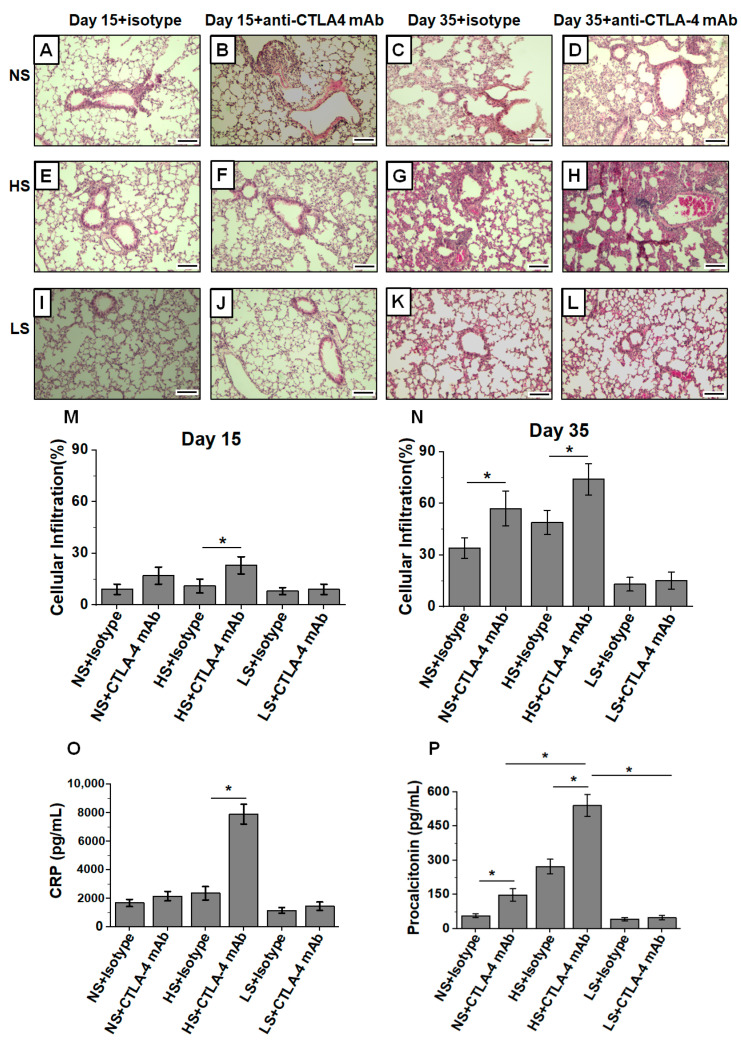
Increased pneumonitis and lung infiltration in tumor-bearing mice on HS diet and treated with anti-CTLA4 mAb based ICI therapy. Lungs are harvested on days 15 and 35 from mice injected with Py230 tumor cells in the mammary fat pad flank region of C57Bl/6 mice and treated with either isotype or anti-CTLA4 mAb i.p. on days 7, 10 and 13. (**A**–**D**) Tumor-bearing mice were fed on NS diet, lungs were harvested on day 15 (isotype, (**A**), and anti-CTLA4 mAb, (**B**)) and day 35 (isotype, (**C**), and anti-CTLA4 mAb, (**D**)). (**E**–**H**) Tumor-bearing mice were fed on HS diet, lungs were harvested on day 15 (isotype, (**E**), and anti-CTLA4 mAb, (**F**)) and day 35 (isotype, (**G**), and anti-CTLA4 mAb, (**H**)). (**I**–**L**) Tumor-bearing mice were fed on LS diet, lungs were harvested on day 15 (isotype, (**I**), and anti-CTLA4 mAb, (**J**)) and day 35 (isotype, (**K**), and anti-CTLA4 mAb, (**L**)). The slides were stained by H&E, and cell infiltration was measured under high power (40×) field. (**M**,**N**) Morphometric analysis for cellular infiltration on day 15 (**M**) and day 35 (**N**). The data are represented as a mean ± SEM for *n* = 6, with each slide read at 5 different high power fields and divided by 5. Scale bar = 200 μm. Statistical analysis performed by one-way ANOVA for multiple comparisons (*) *p*-value < 0.05. (**O**,**P**) Biochemical analysis of inflammatory markers, c-reactive protein (CRP) (**O**) and procalcitonin (**P**) in the blood from various experimental cohorts. Data analyzed by one-way ANOVA for multiple comparisons; data presented as mean ± SEM, *n* = 8 per cohort, (*) *p*-value < 0.05.

As systemic irAE is commonly noticed in ICI therapy, we next determined if irAE is modulated by a salt-modified diet. As shown in Figure 3, there was a markedly increased cellular infiltration into lung epithelial tissue in the anti-CTLA4 mAb-treated HS diet cohort (day 35, Figure 3G,H); however, there was very minimal cellular infiltration in the matched LS diet cohort (day 35, Figure 3K,L). Further, semi-quantitative morphometric analysis demonstrated that (Figure 3M,N), by day 35, following anti-CTLA4 mAb treatment in breasttumor-bearing mice, there was a 3.8 (*p* < 0.05) and 5.1 (*p* < 0.05) fold increase in cellular infiltration into the alveolar epithelium and around terminal bronchioles in the NS and HS diet cohorts, respectively, as compared to the LS diet cohort. An analysis of inflammatory biomarkers (Figure 3O,P), c-reactive protein (CRP) and procalcitonin for systemic inflammatory response demonstrated an enhanced peripheral circulating concentration of these molecules in the HS plus anti-CTLA4 treatment cohort. These data strongly suggest that, upon anti-CTLA4 mAb treatment in breast-tumor-bearing mice, LS diet is associated with decreased systemic inflammatory infiltration to lung and diminished pneumonitis, possibly explaining the survival benefit in this cohort.

### 3.4. Low-Salt Diet Reduced Peripheral Inflammatory Cytokine Response and Alveolar Infiltration with Inflammatory CD4+T Cells following Anti-CTLA4 Therapy

**Figure 4:**
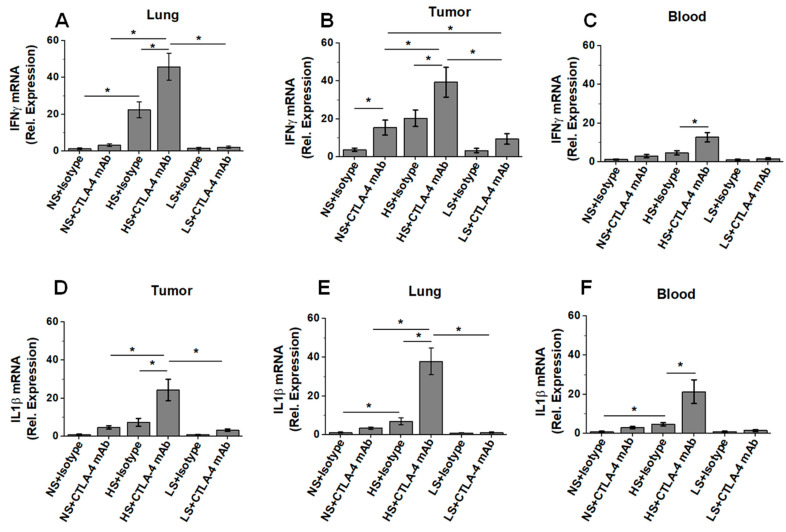
Low-salt diet reduces systemic irAE response in breast-tumor-bearing mice. The mRNA expression of inflammatory cytokines in CD4+T cells were analyzed in tumor-infiltrating, lung-infiltrating and peripheral circulation of breast tumor-bearing mice on ICI therapy following salt diet modification. (**A**–**C**) The mRNA expression of IFNγ in CD4+T cells isolated from tumor (**A**), lung (**B**) and blood (**C**) in NS, HS and LS diet cohorts following treatment with anti-CTLA4 mAb or isotype control. (**D**–**F**) The mRNA expression of IL-1β in CD4+T cells isolated from tumor (**D**), lung (**E**) and blood (**F**) in NS, HS and LS diet cohorts following treatment with anti-CTLA4 mAb or isotype control. Data analyzed by one-way ANOVA for multiple comparisons; data presented as mean ± SEM, *n* = 8 (biological replicates) per cohort, (*) *p*-value < 0.05.

To specifically determine if the decreased lung cellular infiltration in the low-salt-diet cohort was associated with reduced systemic inflammatory irAE response, we initially checked for circulating cytokines levels in the blood of breast-tumor-bearing mice by Luminex. As shown in Table 1, compared to LS diet, HS diet enhanced the systemic circulating levels of inflammatory cytokines IL (interleukin)-1β, TNF (tumor necrosis factor)-α, IFN (interferon)-γ, and IL-17, while reducing the serum levels of anti-inflammatory cytokines such as IL-10. These data suggest that the LS diet significantly reduced the inflammatory immune response. Further, as HS diet is known to induce inflammatory activation of CD4+Tcells, we next determined the inflammatory gene expression (IL1β and IFNγ) in the tumor-infiltrating, lung-infiltrating and circulating CD4+T cells in various salt-diet-modified breast-tumor-bearing mice cohorts isolated by the MACS beads method and with purity ascertained to be >95% by flow cytometry analysis. As shown in Figure 4A, there is a significant increase in the mRNA expression of IFNγ in the tumor-infiltrating CD4+T cells in the HS plus anti-CTLA4 mAb cohort compared to the LS plus anti-CTLA4 mAb cohort (day 35, HS+anti-CTLA4 mAb: 39.4 ± 7.9, LS+anti-CTLA4 mAb: 9.4 ± 2.8 (relative units), *p* < 0.05) and the NS plus anti-CTLA4 mAb cohort (15.4 ± 3.9, *p* < 0.05). Importantly, the LS diet in the anti-CTLA4 mAb-treatment cohort exerted a marked reduction in IFNγ gene expression the lung infiltrating (Figure 4B) and circulating (Figure 4C) CD4+ +T-cells. Further, a similar pattern was observed in the IL1β gene expression in CD4+T-cells (Figure 4D–F). Taken together, these data demonstrated that, upon anti-CTLA4 therapy, the LS diet reduced both pneumonitis and systemic irAE in breast-tumor-bearing mice.

### 3.5. Low-Salt Diet Downregulates NLRP3-Mediated Inflammasome Complex Leading to Diminished irAE

**Figure 5:**
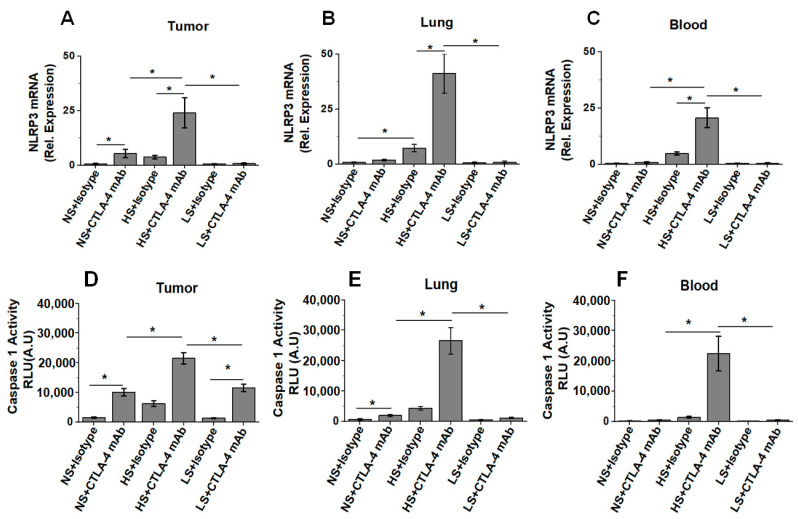
Inflammasome activation in high-salt-diet-fed breast-tumor-bearing mice following anti-CTLA4 mAb therapy. (**A**–**C**) The mRNA expression of NLRP3 in CD4+T cells isolated from tumor (**A**), lung (**B**) and blood (**C**) in NS, HS and LS diet cohorts following treatment with anti-CTLA4 mAb or isotype control. (**D**–**F**) Proteolytic enzyme caspase-1 activity in CD4+T cells isolated from tumor (**D**), lung (**E**) and blood (**F**) in NS, HS and LS diet cohorts following treatment with anti-CTLA4 mAb or isotype control. Data analyzed by one-way ANOVA for multiple comparisons; data presented as mean ± SEM, *n* = 8 (biological replicates) per cohort, (*) *p*-value < 0.05.

As NLRP3-mediated inflammasome activation is well-known to play a key role towards induction of molecular events leading to the enhanced secretion of inflammatory cytokines such as IL-1β [17,43], we next determined if there is an activation of CD4+T cellular inflammasome complex following salt modified diet in breast-tumor-bearing mice. As shown in Figure 5A, NLRP3 gene expression analysis in CD4+T cells demonstrated a 4.5- and 30.1-fold increase in NLRP3 expression levels in the tumor-infiltrating CD4+T cells obtained from the HS diet cohort co-treated with anti-CTLA4 mAb compared to the matched NS (*p* < 0.05) and LS (*p* < 0.05) diet cohorts, respectively. More importantly, the NLRP3 expression in CD4+T cell obtained from lung infiltrating and peripheral circulation (Figure 5B,C) in the high-salt-diet cohort co-treated with anti-CTLA4 mAb demonstrated a 22-fold and 46-fold increase compared to the matched normal-salt (*p* < 0.05) and low-salt (*p* < 0.05) diet cohorts. These data suggest that inflammasome complex is strongly activated by HS diet when co-administered with immune activating checkpoint inhibitor therapy. To further check for the upregulation of downstream events in inflammasome complex, we determined the capase-1 activity by biochemical assay, which is known to play a critical role in the proteolytic cleavage of pro-IL-1β to active secretory form of the inflammatory cytokine, IL-1β. As shown in Figure 5D–F, similar to NLRP3 expression, caspase-1 activity was profoundly increased in the lung infiltrating and circulating CD4+T cells obtained from HS plus anti-CTLA4 cohort. These data clearly support the notion that anti-CTLA4 mAb treatment in breast-tumor-bearing mice put on an HS diet induces the activation of NLRP3-mediated inflammasome complex, leading to systemic irAE response.

### 3.6. Osmosensitive Transcription Factor, NFAT5, Is a Critical Upstream Regulator in Anti-CTLA4 Induced irAE following High-Salt Diet

Previous studies from our laboratory have shown that osmosensitive transcription factor NFAT5 plays a critical role in the inflammatory activation of CD4+T cells following high-salt treatment [33,44]. Towards this, we determined the NFAT5 expression in the tumor-infiltrating CD4+T cells following a salt-modified diet. Initial in vitro high-salt (Δ35 mM NaCl) treatment on naïve CD4+T cells demonstrated enhanced expression of NFAT5, NLRP3 genes along with the upregulation of downstream inflammatory mediators of NLRP3, namely caspase-1 activity and IL1β cytokine gene expression (Supplementary Figure S3). To determine whether NLRP3 is involved in upstream or downstream signaling of NFAT5 following high-salt-mediated hypertonic stress, we performed experiments following specific knock down of NLRP3 by siRNA. As shown in Supplementary Figure S4, NLRP3 knock down did not change the NFAT5 expression; however, it decreased the downstream Caspase-1 activity and IL1β expression. This suggests that NLRP3 is downstream mediated of NFAT5 mediated inflammatory response following high-salt treatment. Next, to determine if NLRP3 is involved in upstream or downstream signaling of NFAT5 following high-salt-mediated hypertonic stress, we performed experiments following the specific knock down of NFAT5 by siRNA. As shown in Supplementary Figure S5, NFAT5 knock down downregulated NLRP3 expression, along with its downstream events, namely caspase-1 activity and IL1β expression. This suggests that NFAT5 is an upstream factor that induces an NLRP3-mediated inflammatory response following high-salt treatment.

**Figure 6:**
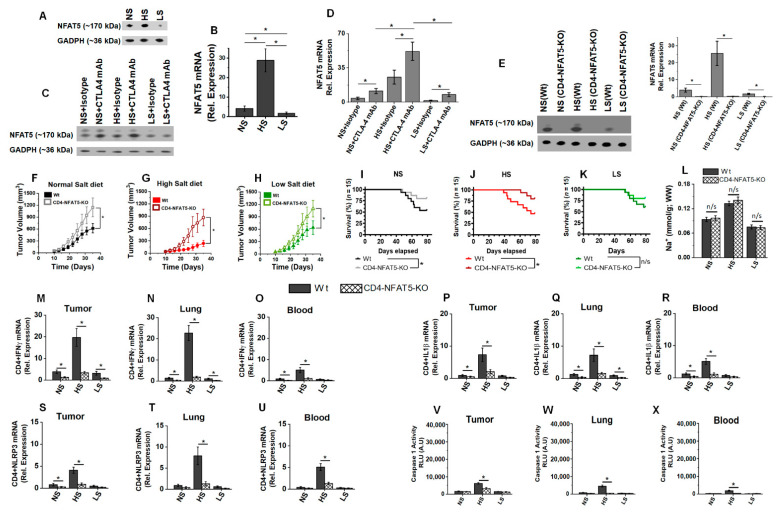
NFAT5 is the upstream modulator of inflammasome activation in high-salt-mediated irAE response. (**A**) Western-blot-based protein analysis for NFAT5 expression in CD4+T cells isolated from tumor-infiltrating immune cells in NS, HS and LS diet cohorts. (**B**) qRT-PCR-based NFAT5 mRNA expression in CD4+T cells isolated from tumor-infiltrating immune cells in NS, HS and LS diet cohorts. (**C**,**D**) Western-blot- (**C**) and qRT-PCR- (**D**) based analysis of NFAT5 expression in CD4+T cells isolated from tumor-infiltrating immune cells in NS, HS and LS diet cohorts following treatment with anti-CTLA4 mAb or isotype control. (**E**) Transgenic murine model with CD4 specific NFAT5 knock out (CD4-NFAT5-KO) in C57Bl/6 background were utilized for further breast tumor studies. Western blot analysis confirmed the lack of NFAT5 expression in our CD4-NFAT5-KO model. (**F**–**H**) Tumor progression kinetics in NS (**F**), HS (**G**) and LS (**H**) cohorts in wild-type (Wt) and CD4-NFAT5-KO transgenic mice injected with syngeneic Py230. Data presented as mean ± SEM, *n* = 8 (biological replicates); statistical analysis was performed using multiple *t*-test, (*) *p*-value < 0.05; n/s = not significant. (**I**–**K**) Survival rate in the three salt-modified diet cohorts—NS (**I**), HS (**J**) and LS (**K**)—in breast-tumor-bearing Wt and CD4-NFAT5-KO mice followed for 80 days. The *n* =15 per cohort, statistical analysis performed by log-rank Mantel–Cox comparison. (**L**) CD4-specific knock out of NFAT5 did not change the tumor [Na^+^] accumulation. (**M**–**O**) The mRNA expression of IFNγ in CD4+T cells isolated from tumor (**M**), lung (**N**) and blood (**O**) of Wt and CD4-NFAT5-KO in NS, HS and LS diet cohorts. (**P**–**R**) The mRNA expression of IL1β in CD4+T cells isolated from tumor (**P**), lung (**Q**) and blood (**R**) of Wt and CD4-NFAT5-KO in NS, HS and LS diet cohorts. (**S**–**U**) The mRNA expression of NLRP3 in CD4+T cells isolated from tumor (**S**), lung (**T**) and blood (**U**) of Wt and CD4-NFAT5-KO in NS, HS and LS diet cohorts. (**V**–**X**) Caspase-1 activity CD4+T cells in tumor (**V**), lung (**W**) and blood (**X**) from Wt and CD4-NFAT5-KO in NS, HS and LS diet cohorts. Data analyzed by one-way ANOVA for multiple comparisons; data presented as mean ± SEM, *n* = 8 (biological replicates) per cohort, (*) *p*-value < 0.05.

In line with this in vitro evidence, our murine studies have also demonstrated that (Figure 6A,B) NFAT5 expression in tumor-infiltrating CD4+T cells was increased in the HS diet cohort more than in the NS and LS diet cohorts. The expression of NFAT5 (Figure 6C,D) was further enhanced upon co-treatment with anti-CTLA4 mAb in the HS diet cohort. To specifically test if NFAT5 plays a direct role in the HS-induced inflammasome-mediated inflammatory activation of CD4+T cells, we utilized a CD4-specific NFAT5 knock out murine model (CD4^cre/cre^TonEBP^flox/flox^C57Bl/6, referred as CD4-NFAT5-KO, Figure 6E and Supplementary Figure S6). As shown in Figure 6F,H, there was enhanced breast tumor progression in CD4-NFAT5-KO mice, suggesting that NFAT5 knock out abrogated the inflammatory anti-tumor activation of CD4+T cells. There was improved survival in NS and HS cohorts (Figure 6I–K) in transgenic breast-tumor-bearing mice compared to wild-type mice. This suggests that the CD4-specific NFAT5 knock out abrogated the systemic irAE response. Of note, the tumor sodium accumulation was similar between transgenic and wild-type tumors, indicating that the change in tumor kinetics and survival was not due to aberrations in tumor sodium localization capability. As it can be noted in Figure 6M–X, the HS diet induced inflammatory activation of CD4+T cells (IFNγ+ and IL1β+), and NLRP3 complex activation was abrogated in CD4-NFAT5-KO transgenic tumor models. These data demonstrated that NFAT5 played a critical role in the HS-mediated inflammatory activation of CD4+T cells and its eventual irAE response.

**Figure 7:**
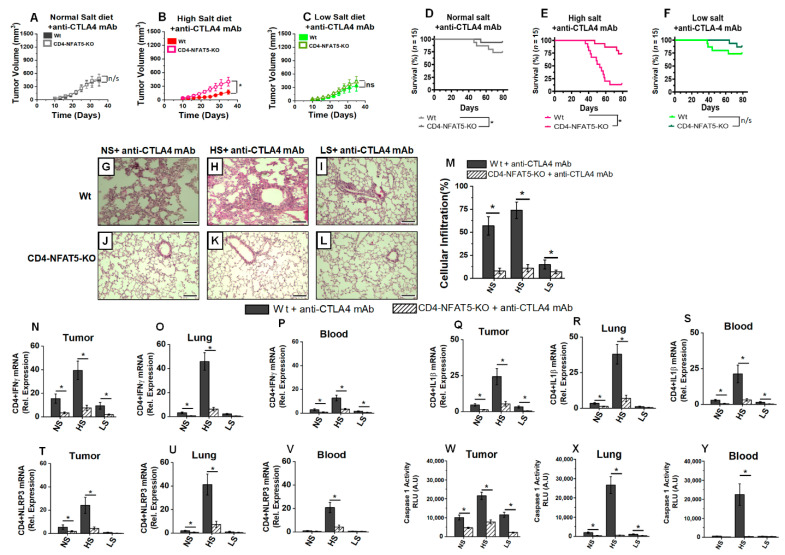
NFAT5 knock out reduces high-salt-mediated inflammatory activation of CD4+T cells and systemic inflammatory response in breast-tumor-bearing mice. (**A**–**C**) Tumor progression kinetics in NS (**A**), HS (**B**) and LS (**C**) cohorts in wild-type (Wt) and CD4-NFAT5-KO transgenic mice injected with syngeneic Py230 following treatment with anti-CTLA4 mAb. Data presented as mean ± SEM, *n* = 8, statistical analysis was performed using multiple *t*-test, (*) *p*-value < 0.05, n/s = not significant. (**D**–**F**) Survival rate in the three salt-modified diet cohorts—NS (**D**), HS (**E**) and LS (**F**), in breast tumor-bearing Wt and CD4-NFAT5-KO mice following treatment with anti-CTLA4 mAb. The *n* =15 per cohort, statistical analysis performed by log-rank Mantel–Cox comparison. (**G**–**L**) Tumor-bearing mice were fed on NS (**G**,**J**), HS (**H**,**K**) and LS (**I**,**L**) diet in breast-tumor-bearing Wt and CD4-NFAT5-KO mice; lungs were harvested on day 35 and analyzed by H&E staining for cellular infiltration. (**M**) Quantitative morphometric analysis of the cellular infiltration in lungs harvested on day 35 from Wt and CD4-NFAT5-KO tumor-bearing mice following treatment with anti-CTLA4 mAb. The data are represented as mean ± SEM for *n* = 6; scale bar = 200 μm. (**G**–**M**), with each slide read at 5 different high-power fields and divided by 5. Statistical analysis performed by one-way ANOVA for multiple comparisons (*) *p*-value < 0.05. (**N**–**P**) The mRNA expression of IFNγ in CD4+T cells isolated from tumor (**N**), lung (**O**) and blood (**P**) of Wt and CD4-NFAT5-KO in NS, HS and LS diet cohorts following treatment with anti-CTLA4 mAb. (**Q**–**S**) The mRNA expression of IL1β in CD4+T cells isolated from tumor (**Q**), lung (**R**) and blood (**S**) of Wt and CD4-NFAT5-KO in NS, HS and LS diet cohorts following treatment with anti-CTLA4 mAb. (**T**–**V**) The mRNA expression of NLRP3 in CD4+T cells isolated from tumor (**T**), lung (**U**) and blood (**V**) of Wt and CD4-NFAT5-KO in NS, HS and LS diet cohorts following treatment with anti-CTLA4 mAb. (**W**–**Y**) Caspase-1 activity CD4+T cells in tumor (**W**), lung (**X**) and blood (**Y**) from Wt and CD4-NFAT5-KO in NS, HS and LS diet cohorts following treatment with anti-CTLA4 mAb. Data analyzed by one-way ANOVA for multiple comparisons; data presented as mean ± SEM, *n* = 8 (biological replicates) per cohort (**N**–**Y**), (*) *p*-value < 0.05.

Next, we wanted to determine if NFAT5 knock out reduced the anti-CTLA4-mediated anti-tumor activation of CD4+T cells. Interestingly, there was no significant difference in the tumor progression kinetics (Figure 7A–C) upon anti-CTLA4 treatment in wild-type and CD4-NFAT5-KO transgenic mice in NS and LS diet cohorts. However, importantly, within the CD4-NFAT5-KO transgenic mice, compared to no treatment (Figure 6F–H), treatment with anti-CTLA4 (Figure 7A–C) significantly reduced the tumor progression kinetics in all three salt modified diet cohorts. These data suggested that anti-CTLA4-mediated anti-tumor activation of CD4+T cells was relatively independent of HS-induced NFAT5 mediated systemic activation of CD4+T cells. This notion was further supported by the improved survival rate (Figure 7D–F) in transgenic mice on NS and HS diet plus anti-CTLA4 treatment. A histological examination of lungs harvested from these various cohorts (Figure 7G–M) demonstrated that, following treatment with anti-CTLA4, there was no significant inflammatory infiltration in the transgenic-tumor-bearing mice from all three diet cohorts, indicating that there was no significant salt-mediated systemic inflammatory response in NFAT5-transgenic mice. This assertion was further supported by the cytokine analysis (Figure 7N–S), wherein we observed reduced IL1β expression in CD4+T cell obtained from tumor, lungs and peripheral blood. Additionally, it is important to note that there was reduced NLRP3/caspase-1 complex activity (Figure 7T) in the transgenic tumor-bearing mice upon treatment with anti-CTLA4 in all three cohorts. The innate immune-activating NLRP3-mediated inflammatory response is generally considered rather non-tumor-specific and generalized. Our data suggested that, while anti-CTLA4-mediated-specific anti-tumor response (Figure 7N,Q) was maintained by NFAT5 knock out, non-tumor-specific (generalized) salt-mediated NLRP3 pro-inflammatory responses were abrogated by NFAT5 knock out (Figure 7T,W). Furthermore, as noted in Figure 7U,V,X,Y, NLRP3 activity was downregulated in lung and peripheral circulating CD4+T cells, suggesting that NFAT5 knock out abrogated HS-mediated systemic inflammatory responses. Taken together, all these data strongly suggest that, following HS diet, NFAT5 upregulation leads to the systemic inflammatory activation of CD4+T cells, which is further enhanced by immune check point therapy, leading to systemic inflammatory response and poor survival outcomes. Importantly, LS diet, on the other hand, reduced the systemic inflammatory irAE response while retaining the anti-tumor efficacy of anti-CTLA4 therapy.

## 4. Discussion

The selective accumulation of sodium in solid organ tumors along with its pathophysiological role is an area of intense interest in cancer research. An enhanced sodium accumulation has been shown in solid organ tumors such as breast, prostate and brain [4,5] compared to surrounding soft tissue [6,7,8]. Although the exact molecular basis of this sodium accumulation in solid organ tumors is still unknown, it can be envisioned that the probable cause could be high levels of extracellular matrix proteins with acidic amino acid residues in solid organ tumors produced by cancer-associated fibroblasts. In line with this evidence, our current sodium analysis using Na^23^-MRI and quantitative flame absorbance (Figure 1) demonstrated high sodium concentration in murine breast tumors, as compared to contralateral mammary fat pad. Interestingly, the HS diet further enhanced intra-tumor sodium accumulation, while the LS diet reduced intra-tumor sodium accumulation to levels almost similar to those in surrounding soft tissues, thus suggesting that intra-tumor sodium accumulation could be modulated by a salt-modified diet. In general, potassium concentration is higher inside the cell, while sodium concentration is higher outside the cell. Further, 90–95% of ionic channels on the cell membrane are potassium channels. It is important to note that, while the concentration of potassium in the LS cohort (Figure 1G) is high, the tumor osmolality (Figure 1E) did not increase in the LS cohort. Further, the sodium concentration in the LS tumors was low compared to that in the HS cohort (Figure 1F). This suggested to us that the potassium concentration increase in LS tumors did not induce osmotonic stress. We think the increased potassium concentration could be due to a combination of two events: (i) in LS diet tumors, to compensate for the apparent decreased influx of sodium ions (from outside to inside) the tumor cell, these cells could downregulate potassium channels to reduce potassium efflux (from inside to outside) across the cell membrane to maintain enough cations needed for physiologic resting membrane potential; (ii) as the intracellular concentration of potassium is high, an increased number of tumor cells in the LS cohort (compared to the HS cohort) could mean a higher potassium concentration in our ionic analysis. However, this hypothesis should be tested in the future with detailed membrane biophysics experiments.

The American Heart Association recommends a daily dietary sodium intake of 1.5 g, which would be equivalent to 3.75 g per day of table salt (NaCl) [45]. However, it is considered that an average adult human in the US consumes 9 to 12 g of salt per day [46]. A high-salt diet is a well-established risk factor for cardiac, vascular, autoimmune and chronic inflammatory diseases [47,48,49]. Although a direct correlation between high-salt diet and human breast cancer is not readily available in the scientific literature, there are some apparently contradictory findings on the relationship between salt diet and gastric cancer in human versus pre-clinical models [50,51,52]. Human studies by Wilck et al. demonstrated that HS diet induces the depletion of *Lactobacilus*, generally considered good bacteria, in the gut microbiome, leading to the aggravation of autoimmune diseases in a murine model [53]. Consequently, in contrast, studies by Razvi et al. demonstrated that HS diet enhances *Bifidobacterium*, also generally considered good bacteria in the gut microbiome, and caused microbiome-mediated HS-induced anti-tumor response through the activation of natural killer (NK) cells [26]. These data indicate a complicated interaction between the role of salt on multiple variables impacting cancer growth and proliferations. In vitro cell-culture-based studies from our laboratory have shown that high-salt treatment enhances breast cancer cell proliferation through releasing G0/G1 arrest and upregulating mitosis [29,37,54,55,56]. Further, pre-clinical studies by Willebrand et al. [24] and He et al. [25] have demonstrated that a HS diet in murine breast and melanoma tumor models have resulted in diminished tumor progression along with the inhibition of immunosuppressive myeloid-derived suppressor cells (MDSCs). In agreement with these broad outcomes from multiple laboratories, our current evidence also shows that an HS diet inhibits tumor progression kinetics in our murine breast tumor model. All these data can be considered intriguing, yet they are logically expected, in that an HS diet induces the inflammatory activation of immune cells, leading to an anti-tumor response. However, an important question arises on the probable impact of HS diet on the systemic inflammatory response. There is a wide variation in the definition of a salt-modified diet and the timing of starting this diet. For example, Rizvi et al. [26] have defined a low-salt diet as 1% NaCl above a regular salt (0.9% NaCl) diet; He et al. [25], in their studies, have switched their models to HS diet at the time of tumor inoculation; and Willebrand et al. [24] performed a diet switch two weeks before tumor inoculation. It is to be noted that, in our studies, we performed the diet switch 2 weeks prior to tumor inoculation, with the rationale that, usually, human dietary salt (high or low) is a chronic phenomenon, and towards that, murine models should be first stabilized on a salt-modified diet to induce salt-specific pathophysiological changes before the inoculation of tumor cells. Further, our definition of an HS diet is 4% NaCl food with 1% additional supplemented NaCl in drinking water. The LS diet in our experiments refers to 0.1% NaCl food with no additional salt supplement to water which would be sufficient to meet the basic sodium metabolic requirements. Our regular/normal-salt diet would refer to 1% NaCl food with no additional salt supplement to water. Luminex-based multi-cytokine analysis in the peripheral blood of tumor-bearing mice on day 35 (Table 1) demonstrated elevated levels of inflammatory cytokines (IFNγ, IL-1β, TNFα and Il-17) in the HS diet cohort, while there were elevated anti-inflammatory IL-10 blood levels in the LS diet cohort. These data suggested to us that there was a systemic inflammatory response mediated by the HS diet in our murine tumor models. This assertion is further confirmed by the decrease in survival rate (Figure 2) in the HS diet compared to the LS diet cohort, which could be explained by the systemic inflammatory response leading to a higher death rate in the HS diet cohort.

We chose to use Py230-C57Bl/6 breast cancer model, as this is a non-invasive model with very minimal metastasis, unlike the 4T1 breast cancer model, which is a metastatic model with known extensive metastasis to other solid organs [57]. Our choice of this non-metastatic model allowed us to specifically study irAE response in the relative absence of cancer metastatic side-effects. Although both CTLA-4 and PD-1 exert an inhibitory immune response (including impact on tumor kinetics), the mechanism of action and the site of expression are significantly different. CTLA-4 is shown to regulate T-cell proliferation early in an immune response, primarily in lymph nodes, whereas PD-1 suppresses T cells later in an immune response, primarily in the tumor tissues [58]. This could explain the reason for the higher irAE side-effect profile in anti-CTLA4 therapy as compared to anti-PD1 therapy. Anti-CTLA4-based activation begins early and is prolonged, as the impact is from draining lymph nodes. On the other hand, PD1 expression and T-cell impact is in tumor-infiltrating immune cells and tumor microenvironment. PDL1 expression in Py230 is lower compared to 4T1 [57]. The ability of anti-PD1-mediated therapy is heavily dependent upon the tumor load, tumor cell progression phase and tumor expression of PD-L1. This might explain the difference in the survival data between HS diet plus anti-PD1 therapy [25,26] versus our current HS diet plus anti-CTLA4 therapy studies.

Systemic inflammatory response, irAE, following ICI therapy is a major challenge in cancer immunotherapy [59]. Given the well-accepted notion in clinical outcomes that irAE could be considered a positive marker for immunotherapy, the molecular link and precise demarcation between beneficial cancer immunotherapeutic efficacy and unacceptable irAE is yet to be defined [60]. Commonly reported irAE responses in patients on ICI include inflammatory damage to solid organs, such as pneumonitis, myocarditis and colitis [61,62]. Overcoming irAE while retaining cancer immunotherapeutic efficacy is central for ICI success. The anti-CTLA4 mAbs act by blocking the inhibitory signal mediated by the interaction of B7 protein, an integral membrane protein on activated antigen presenting cells, with CTLA4 on naïve T cells to inhibit inflammatory effector activation of T-cells (T_eff_), mainly at the tumor and draining lymph nodal sites [63]. As HS diet is also associated with the inflammatory Th17 and Th1 activation of CD4+T cells [21], we proposed to test a hypothesis if combination of HS diet with anti-CTLA4 mAb induces irAE response. Our data demonstrated that HS diet enhanced pneumonitis (Figure 3) and enhanced inflammatory cytokine (IFNγ and IL-1β) expression in CD4+T cells in sites distal to tumor such as lung and also peripheral circulation (Figure 4). Canonical NLRP3-inflammasome complex activation is associated with several systemic inflammatory responses [64]. Our data demonstrated that HS diet coupled with anti-CTLA4 mAb induced an enhanced NLRP3 expression in CD4+T cells in systemic circulation. Previous studies from our laboratory and others have demonstrated that osmosensitive transcription factor NFAT5 upregulation played a critical role in the HS-diet-mediated inflammatory activation of CD4+T cells [33]. In agreement, our current studies demonstrate that NFAT5 knock out in CD4+T cells abrogated (Figure 6) HS-diet-mediated systemic inflammatory responses. Taken together, all these observations strongly suggest that HS diet induces NFAT5/NLRP3 inflammasome complex upregulation, resulting in CD4+T-cell-mediated pro-inflammatory systemic irAE response.

Interestingly, our studies revealed that a low-salt diet decreased the peripheral frequency of inflammatory cytokine expressing CD4+T cells. This led us to hypothesize that an LS diet would reduce irAE response following anti-CTLA4 mAb therapy. Compared to the isotype control, the low-salt diet, when combined with anti-CTLA4 mAb, induced significant inhibition in tumor progression kinetics, thus suggesting that the LS diet did not impact the inflammatory activation of tumor infiltrating CD4+T cells. It is important to note that (Figure 4), in the LS cohort, following anti-CTLA4 mAb therapy, compared to the isotype control, while the frequency of tumor infiltrating inflammatory CD4+T cells was high, there was no significant increase in the inflammatory phenotype in the lung and peripheral circulation. Thus, we conclude that LS diet plus anti-CTLA4 mAb therapy exerted anti-cancer immunotherapeutic efficacy, improved the survival rate, and, at the same time, reduced the systemic irAE response.

Clinical studies have shown that, compared with anti-PD1 blockade, anti-CTLA4 is known to cause higher irAE response [11,65,66,67]. As high as 60% of patients treated with anti-CTLA4 had irAE symptoms of any grade, along with a grade ≥3 irAE in 10–30% of patients [11]. In stage III melanoma patients, a phase III clinical trial with anti-CTLA4 therapy demonstrated that 54.1% of patients developed grade ≥3 irAE symptoms. However, only 10% of patients on anti-PD1 therapy developed irAE symptoms. This clearly points to the key mechanistic difference between the two ICIs (anti-CTLA4 vs. anti-PD1). Further studies are needed to study the impact of LS diet on other (non-CTLA4-based) ICI therapies on cancer immunotherapeutic efficacy and irAE incidence. Further, in the tumor microenvironment, the success of ICI is dependent upon the Treg frequency in tumors and the activation/inhibition of other immune cells such as NK cells and MDSCs. A comprehensive high-throughput-based immune landscape study following the coupling of ICI with an LS diet is needed for an in-depth understanding of the impact of LS diet on cancer immunotherapy. The microbiome has been shown to play a critical role on tumor immune responses. In pre-clinical models, the differences in microbiome between in-house mouse breeding strains and vendor mouse breeding strains could also have an impact, which should be considered in tumor immunotherapy studies. In addition to NFAT5, other pathways such as the serum glucocorticoid kinase 1 (SGK1)- [21,22,23] and salt inducible kinase 3 (SIK3)- [37] mediated mechanism could also play a significant role in salt diets, which needs to be investigated further. Interestingly, chronic metabolic diseases such as diabetes and hypertension were identified to be consequences of ICI therapy [68]. The impact of ICI on metabolic diseases need to be studied in future. All pre-clinical tumor inoculation models (including our current study) have a generic problem of studying tumor responses during an acute immune activation phase [28]. However, human cancers are a consequence of initial immune elimination followed by cancer growth during the immune exhaustion phase. A precise immune exhaustion model probably using humanized PDX models is needed to evaluate the role of salt diet modification on ICI response. In our current study, we specifically focused on CD4+T cells; however, in the future, it is important to study the functionality of other immune cells such as cytotoxic CD8+T cells, MDSCs, etc., to check for the low-salt-diet-mediated attenuation of the irAE response.

## 5. Conclusions

In summary, we showed that an HS diet in combination with anti-CTLA4 mAb enhanced systemic irAE response. On the other hand, an LS diet significantly reduced the irAE response following anti-CTLA4 mAb therapy in our breast-cancer-based preclinical murine model. Although the role of a low-salt diet in clinical cancer patients is unknown, our data suggest that a low-salt diet, through the downregulation of NFAT5-mediated inflammasome complex, reduces pneumonitis and systemic circulating frequency pro-inflammatory CD4+T cells. Our current data could provide the basis for future clinical studies for the potential low-salt dietary recommendation to cancer patients to overcome irAE while retaining cancer immunotherapeutic efficacy.

## Figures and Tables

**Table 1:** Expression of cyto/chemokines in the blood (day 35) from breast-tumor-bearing mice following salt modified diet (NS, HS and LS). All experiments were performed in four independent biological replicates and protein values were presented as mean ± SEM, (*) *p*-value < 0.05 (ANOVA one-way post hoc Bonferroni correction, compared with NS cohort), (^$^) *p*-value < 0.05 (ANOVA one-way post hoc Bonferroni correction, compared with HS cohort).

	Regular Diet	High Salt	Low Salt
Effector Cytokines			
IL-1β	427 ± 111	1453 ± 256 *	203 ± 89 *^,$^
TNF-α	511 ± 93	1818 ± 279 *	367 ± 83 *^,$^
IFN-γ	342 ± 77	1507 ± 301 *	312 ± 84 *
IL-17A	27 ± 4	79 ± 13 *	n.d.
Anti-inflammatory Cytokines			
IL-4	n.d.	n.d.	n.d.
IL-10	3659 ± 394	1018 ± 238 *	5138 ± 651 *^,$^
Immunostimulatory Cytokines			
Eotaxin	34 ± 12	49 ± 17	33 ± 18
IL-1α	n.d.	7.8 ± 3.6	n.d.
IL-2	6.2 ± 4.9	6.6 ± 3.1	5.9 ± 2.7
IL-5	n.d.	n.d.	n.d.
IL-6	216 ± 68	582 ± 91 *	114 ± 73 ^$^
IL-12(p40)	673 ± 116	803 ± 132	754 ± 82
IL-12(p70)	256 ± 59	298 ± 102	272 ± 73
IL-13	12.1 ± 2.7	39.4 ± 8.3 *	11.9 ± 3.7
Chemoattractants			
RANTES	1042 ± 287	2365 ± 397 *	1246 ± 231 ^$^
MIP-1α	127 ± 37	496 ± 82 *	94 ± 21 ^$^
MIP-1β	214 ± 47	726 ± 54 *	376 ± 97 ^$^
MCP-1	184 ± 39	212 ± 42	206 ± 58
KC	17 ± 4	15 ± 5	22 ± 7
Growth Factors/Cell differentiation factors			
IL-9	n.d.	n.d.	n.d.
IL-3	2673 ± 538	3982 ± 537 *	698 ± 269 *^,$^
GM-CSF	22 ± 7	38 ± 9	32 ± 9
KC	17 ± 4	15 ± 5	22 ± 7
G-CSF	513 ± 97	583 ± 104	638 ± 96

## Data Availability

All data used in this article is provided in the body or in supplementary figures.

## Supplementary Materials

The following supporting information can be downloaded at: https://www.mdpi.com/article/10.3390/biology11060810/s1, Figure S1: Representative flow cytometry analysis of CD4+T cell purity following MACS bead isolation from tumor and blood; Figure S2: Percent Weight change from baseline in salt modified diet cohorts following treatment with anti-CTLA4 mAb or isotype control; Figure S3: High salt (Δ35 mM NaCl) mediated in vitro inflammatory activation of CD4+T cells; Figure S4: NFAT5 mediated upregulation of NLRP3 inflammatory responses in CD4+T cells following in vitro high salt (Δ35 mM NaCl) treatment; Figure S5: NFAT5 mediated upregulation of NLRP3 inflammatory responses in CD4+T cells following in vitro high salt (Δ35 mM NaCl) treatment; Figure S6: Densitometry analysis of the Western blots shown in Figure 6A,C,E, respectively; Figure S7: Original Gels.
