# Supplementary material for: Low-Salt Diet Reduces Anti-CTLA4 Mediated Systemic Immune-Related Adverse Events while Retaining Therapeutic Efficacy against Breast Cancer
Source: Biology (Basel). 2022 May 25;11(6):810. doi: 10.3390/biology11060810 (PMC9219826; doi:10.3390/biology11060810)
Supplement: Supplementary file 1 [file biology-11-00810-s001.zip › biology-1643122 - Supplementary.pdf]

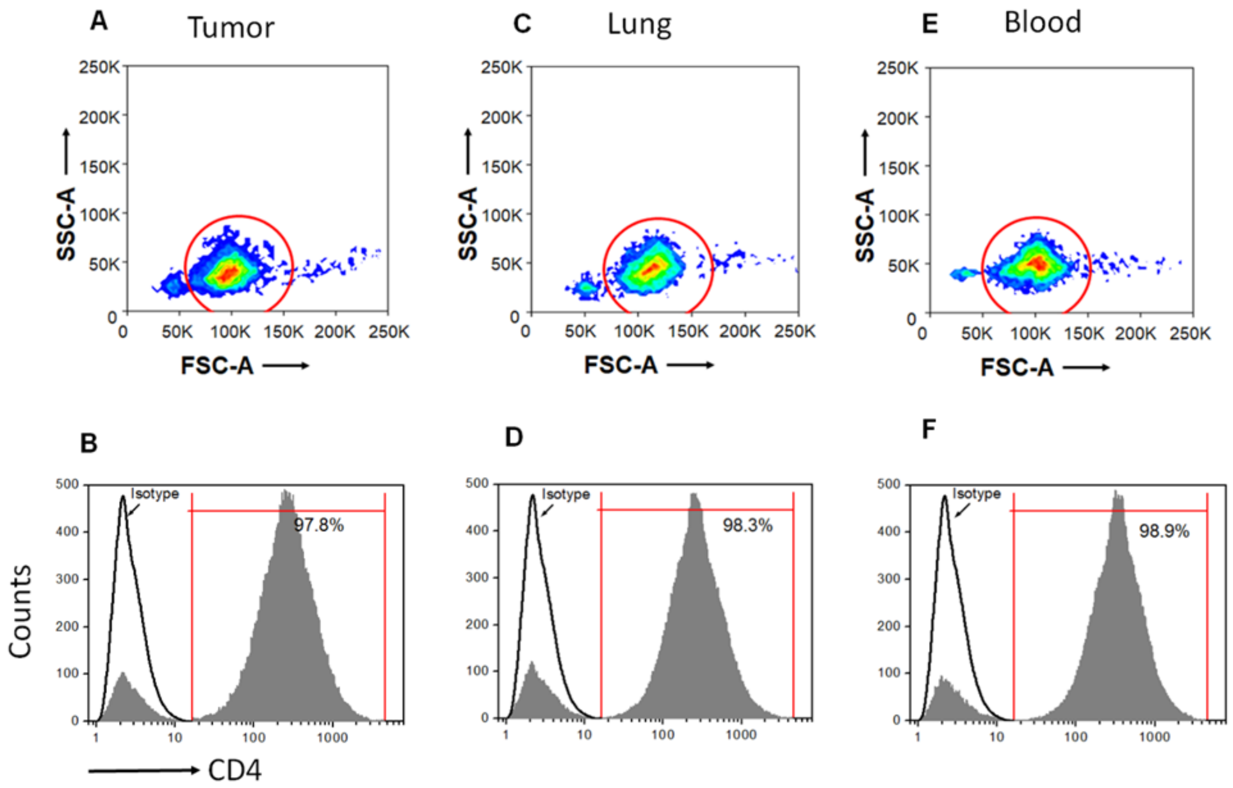

Figure S1. Representative flow cytometry analysis of CD4+T cell purity following MACS bead isolation from tumor (A, B), Lung (C,D) and blood (E,F). In the FSC/SSC plot, lymphocytes were shown in the red gated region (A,C,E) of our MACS beads isolated cells. The lymphocyte gated population was >95% in all our magnetic bead isolations (from tumor, lung and blood). The CD4+T cell isolations from three tissues (B, D, E) with a purity >95% only were utilized for functional and molecular studies performed in our experiments.

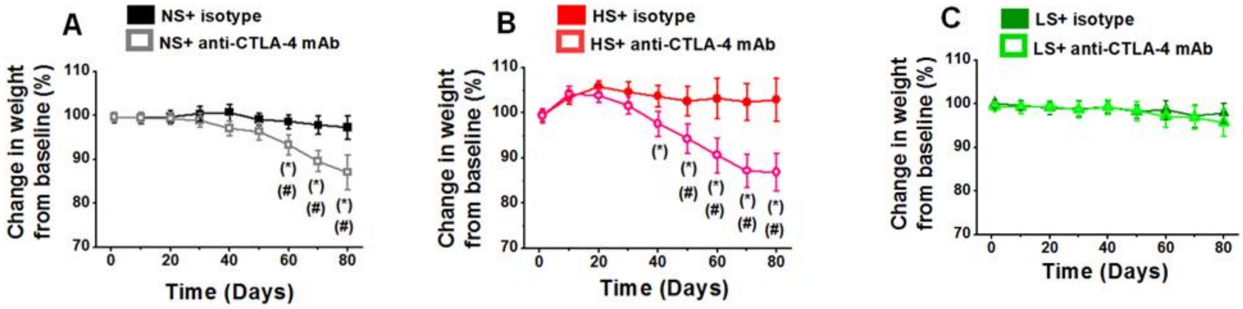

Figure S2. Percent Weight change from baseline in salt modified diet cohorts following treatment with anti-CTLA4 mAb or isotype control. The weight change kinetics in NS (A), HS (B) and LS (C) cohorts following treatment with anti-CTLA4 monoclonal antibody (mAb) or isotype control with 200 μg injected intraperitoneally (i.p.) on days 7, 10 and 13, and followed for 5 weeks. All cohorts were started on day 1 with $n = 15$ (same cohorts used in figure 2). Signed rank tests with step-down Sidak adjustment was used for comparisons of weight from day 1 to subsequent time points as determined by significance $p < 0.05$; (#)significant difference between day 1 and individual time point. Wilcoxon-Mann Whitney tests for comparisons of weights between cohorts at given time point (*) significance determined by $p < 0.05$.

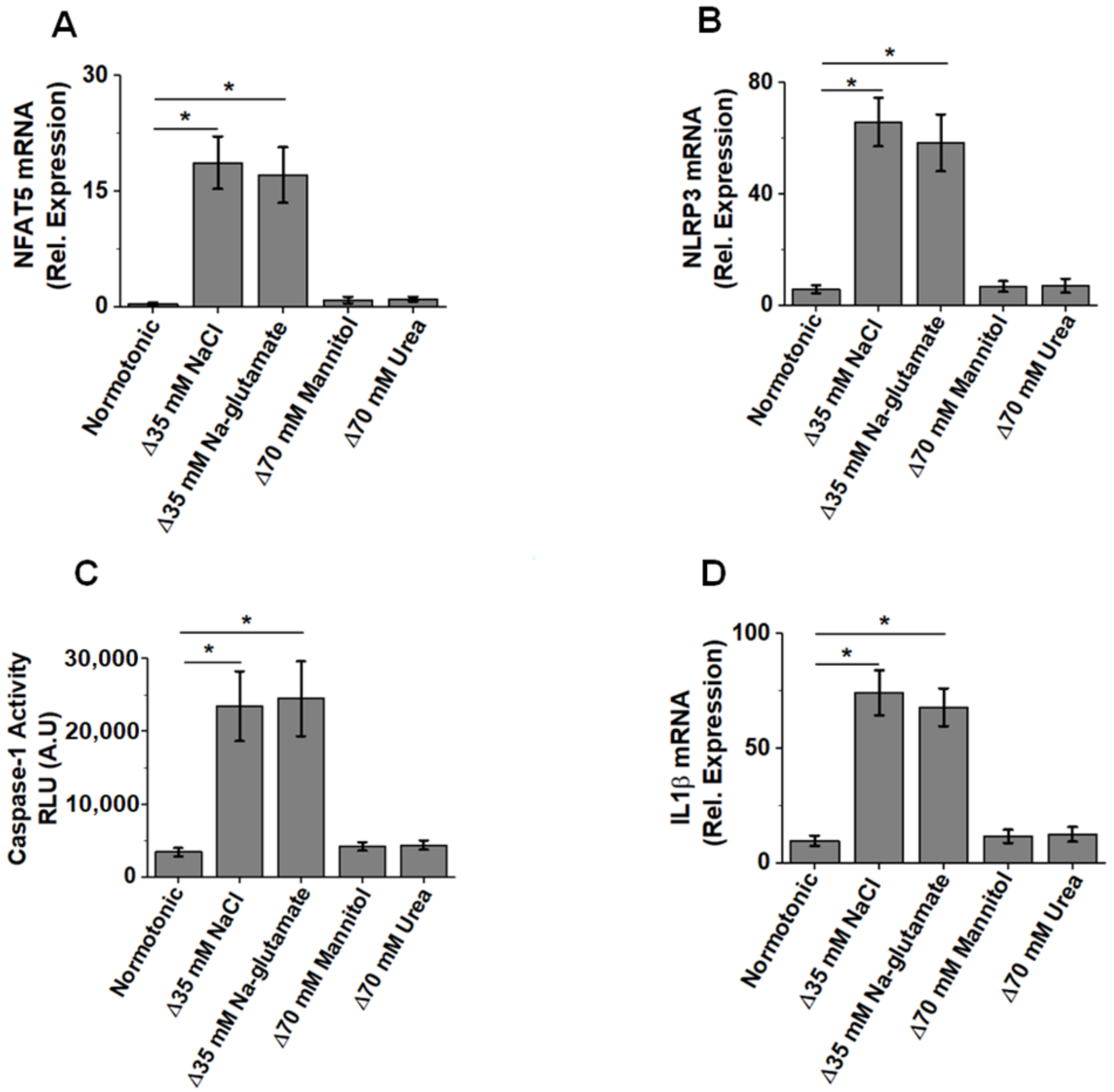

Figure S3. High salt (Δ35 mM NaCl) mediated in vitro inflammatory activation of CD4+T cells. Previous studies in our laboratory have demonstrated that (PMID: 33918403) that 35 mM NaCl induced NFAT5 mediated ex vivo inflammatory activation and Th1/Th17 phenotype switch of CD4+T cells. In our current study, we determined if similar hypertonic salt induces NLRP3 activation. Towards this, naïve CD4+T cells were isolated from splenocytes by MACS beads and purity assessed by flow cytometry to be >95% (described in methods section). Each in vitro stimulation lasted for 7 days, which consisted of treatment with 35 mM NaCl (or 35 mM sodium glutamate), anti-CD3 monoclonal antibody (mAb, 2 μg/mL) CD28 mAb (2 μg/mL) for 3 days, followed by addition of IL-7 (10 ng/mL), and IL-2 (20 ng/mL). Osmotic/ionic equimolar (70 mM) mannitol or urea was used instead of NaCl as controls in our experiments. The mRNA expression of NFAT5 (A) and NLRP3 (B) was determined. The downstream activity of NLRP3 complex was assessed by Caspase-1 activity (C) and IL1β mRNA expression. Data presented as mean ± standard error of mean (SEM) from 5 independent experiments. Data analyzed by one-way ANOVA for multiple comparisons, (*) $p < 0.05$.

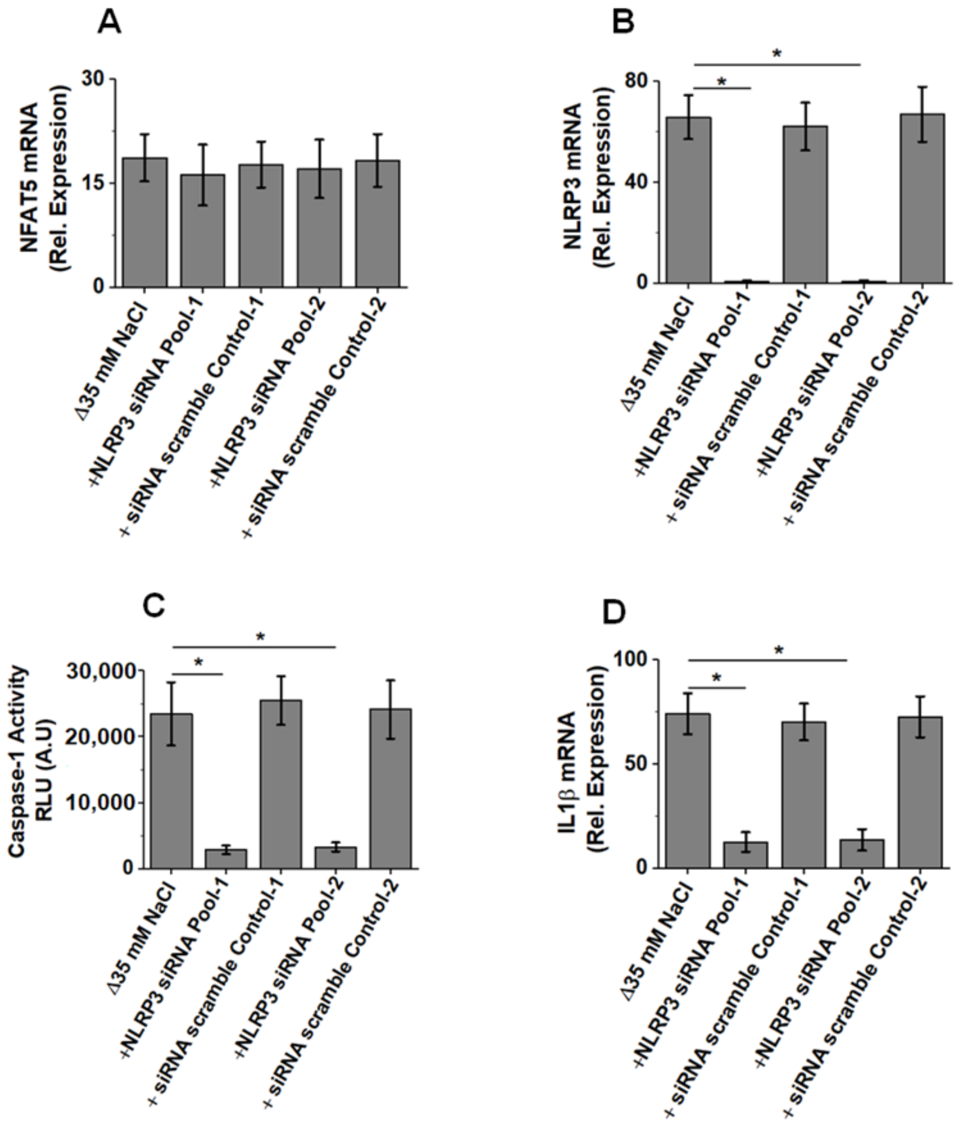

Figure S4. NFAT5 mediated upregulation of NLRP3 inflammatory responses in CD4+T cells following in vitro high salt (Δ35 mM NaCl) treatment. To determine if NLRP3 is involved in upstream or downstream signaling of NFAT5 following high salt mediated hypertonic stress we have performed experiments following specific knock down of NLRP3 by siRNA. Two pooled NLRP3 specific siRNA were obtained from two independent vendors. In pooled-1 experiments, cells were transfected with either with mouse NLRP3 siRNA (Accel SMARTpool set of 4 siRNA, final concentration, 25 nM; cat. EQ-053455-00-0020, Dharmacon, Lafayette, CO, USA) or scrambled control siRNA (cat. D-001910-10-50, Dharmacon, Lafayette, CO, USA) were performed by using Accell siRNA reagents provided by manufacturer. In pooled-2 experiments, cells were transfected by either with mouse NLRP3 siRNA with a pool of 3 target-specific 19-25 nt siRNAs designed to knock down gene expression (Cat# sc-45470, final concentration, 25 nM, Santa Cruz Biotech, Dallas, TX, USA) or scrambled control siRNA (sc-37007) were performed by using manufacturer provided siRNA transfection Reagent (sc-29528), transfection medium (sc-36868) and dilution buffer (sc-29527). The mRNA expression of NFAT5 (A) and NLRP3 (B) was determined. The downstream activity of NLRP3 complex was assessed by Caspase-1 activity (C) and IL1β mRNA expression. Data presented as mean ± standard error of mean (SEM) from 5 independent experiments. Data analyzed by one-way ANOVA for multiple comparisons, (*) $p < 0.05$. As shown in this figure, NLRP3 knock down did not change the NFAT5 expression, however, decreased the downstream Caspase-1 activity and IL1β expression. This suggests that NLRP3 is

downstream mediated of NFAT5 mediated inflammatory response following high salt treatment.

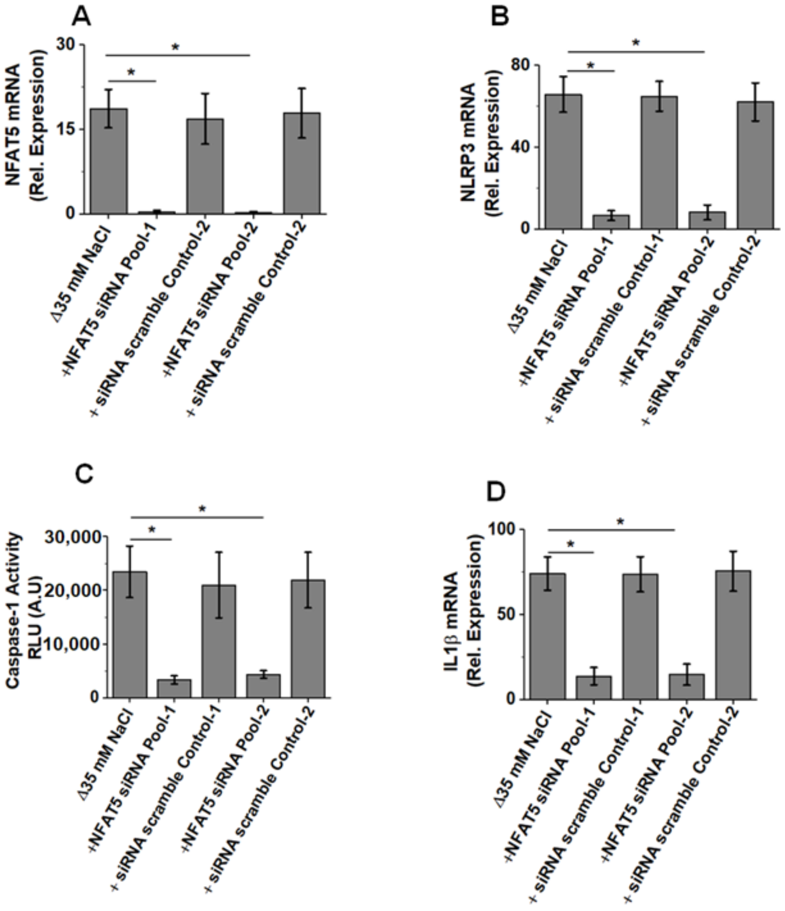

Figure S5. NFAT5 mediated upregulation of NLRP3 inflammatory responses in CD4+T cells following in vitro high salt (Δ35 mM NaCl) treatment. To determine if NLRP3 is involved in upstream or downstream signaling of NFAT5 following high salt mediated hypertonic stress we have performed experiments following specific knock down of NFAT5 by siRNA. Two pooled NFAT5 specific siRNA were obtained from two independent vendors. In pooled-1 experiments, cells were transfected with either with mouse NFAT5 siRNA (Accel SMARTpool set of 4 siRNA, final concentration, 25 nM; cat. EQ-058868-00-0020, Dharmacon, Lafayette, CO, USA) or scrambled control siRNA (cat. D-001910-10, Dharmacon, Lafayette, CO, USA) by using Accell siRNA reagents provided by manufacturer. In pooled-2 experiments, cells were transfected by either with mouse NFAT5 siRNA with a pool of 3 target-specific siRNAs for knock down (Cat# sc-38122, final concentration, 25 nM, Santa Cruz Biotech, Dallas, TX, USA) or scrambled control siRNA (sc-37007x) were performed by using manufacturer provided reagents.The mRNA expression of NFAT5 (A) and NLRP3 (B) was determined. The downstream activity of NLRP3 complex was assessed by Caspase-1 activity (C) and IL1β mRNA expression. Data presented as mean ± standard error of mean (SEM) from 5 independent experiments. Data analyzed by one-way ANOVA for multiple comparisons, (*) $p < 0.05$. As shown in this figure, NFAT5 knock down downregulated NLRP3 expression, along with its downstream events, namely, caspase-1 activity and IL1β expression. This suggests that NFAT5 is upstream factor towards inducing NLRP3 mediated inflammatory response following high salt treatment.

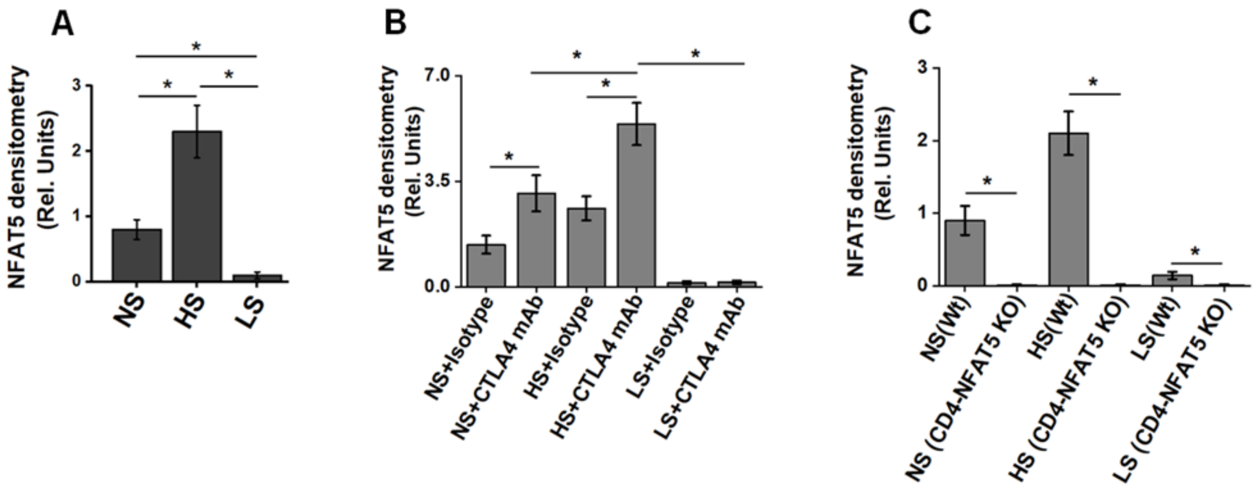

Figure S6. (A-C) Densitometry analysis of the Western blots shown in figure 6 A, C and E, respectively. Data presented as mean ± SEM, n=8 (biological replicates), statistical analysis was performed using multiple t-test, (*) p-value < 0.05.

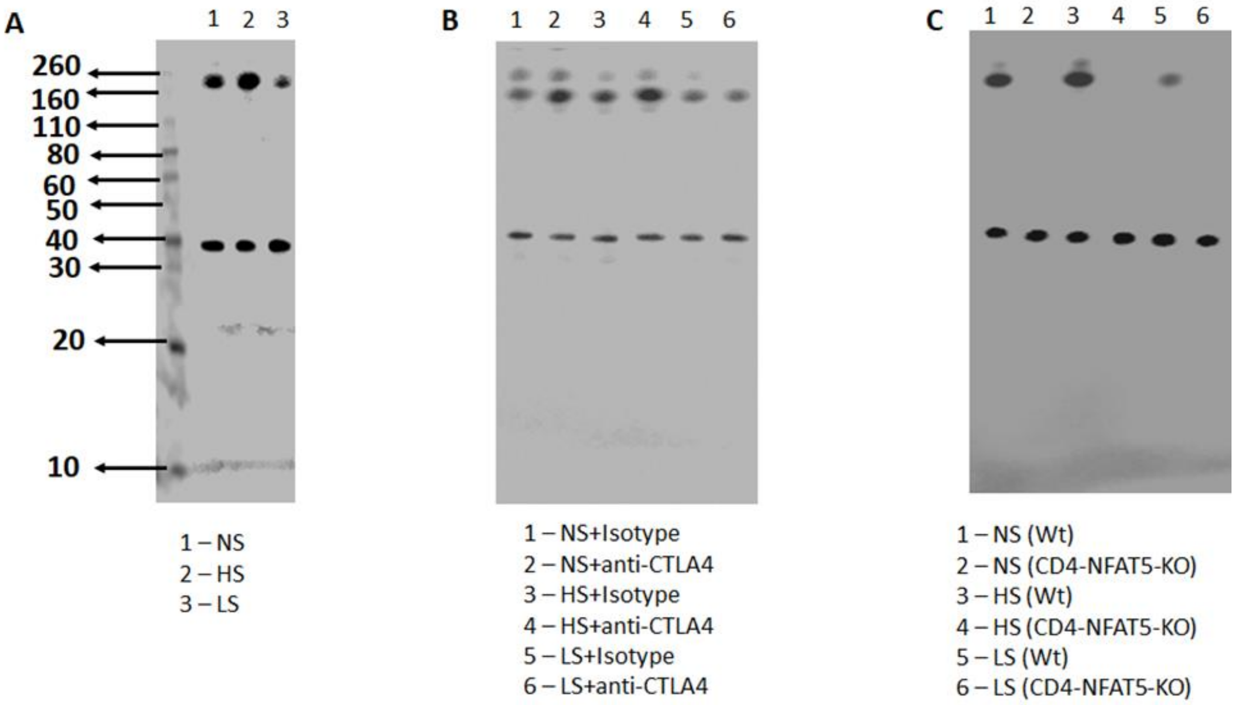

Figure S7. Original Gels: (A) full gel for figure 6A; (B) full gel for figure 6C; and (C) full gel for figure 6E. Primary Antibody: mouse monoclonal NFAT5 (sc-398171, Santa Cruz) -1:1000 dilution (and) mouse monoclonal GADPH (sc-32233, Santa Cruz) -1:1000 dilution; Secondary Antibody: goat anti-mouse-HRP(sc-2005, Santa Cruz) - 1:5000 dilution.
